# Easy scalable, low-cost open-source magnetic field detection system for evaluating low-field MRI magnets using a motion-tracked robot

**DOI:** 10.1007/s10334-025-01239-1

**Published:** 2025-04-05

**Authors:** Pavel Povolni, Robin Bendfeld, Sergej Maltsev, Judith Samlow, Felix Glang, Praveen Iyyappan Valsala, Dominique Goerner, Dario Bosch, Sebastian Mueller, Florian Birk, Kai Buckenmaier, Klaus Scheffler

**Affiliations:** 1 High‑Field Magnetic Resonance Center, Max Planck Institute for Biological Cybernetics, 72076 Tübingen, Germany; 2 Institute for Nonlinear Mechanics, Department of Mechanical Engineering, University of Stuttgart, 70569 Stuttgart, Germany; 3 Department for Biomedical Magnetic Resonance, University of Tübingen, 72076 Tübingen, Germany

**Keywords:** Low-field magnetic resonance imaging, Open-source hardware, Magnetic field mapping, Robotics, Low-cost hardware

## Abstract

**Objective:**

Low-field magnetic resonance imaging is currently developing into a valuable diagnostic tool due to its simplicity of magnet array designs. Particularly, this allows the development of scanners as part of educational workshops, thus ensuring knowledge transfer and empowering local scientists to design tailored solutions for specific local problems. To obtain the maximum performance, the magnet needs to be shimmed requiring an automated system measuring the spatial magnetic field distribution.

**Methods:**

A self-designed measuring probe based on commercial integrated Hall sensor chips is used and optimized by calibrating it in an easy-to-build calibration system. For positioning of the sensor, a low-cost five-degree-of-freedom robot arm is used and improved by camera-based motion tracking for precise localization of the sensor.

**Results:**

The system is able to map the field of a $45\text{mT}$-Halbach desktop MR magnet, as well as a self-designed x-gradient (used inside the magnet) with an efficiency of $2\text{mT}/\text{m}/\text{A}$. The built-up Hall sensor demonstrates a level of precision that is competitive with commercial sensors. The entire positioning system can be freely scaled to accommodate larger designs by adjusting the kinematics.

**Conclusion:**

The presented system is demonstrated to be comparable to already established measurement systems, while the costs, setup times, and mapping duration are greatly reduced.

**Supplementary Information:**

The online version contains supplementary material available at 10.1007/s10334-025-01239-1.

## Introduction

Magnetic resonance imaging (MRI) became an important instrument in medical diagnostics and science, having evolved significantly since the first devices were introduced in the 1970s [1]. The development of stronger magnetic fields and advancements of hardware (e.g., power amplifier and antennas) and software (e.g., sequences and reconstruction methods) have contributed to the improvement of image quality, thus establishing MRI as a key medical device in industrialized countries, with over 100 million scans performed annually [2]. However, the acquisition, installation, and service of these machines requires enormous financial resources, which has resulted in limited access to this technology in low- and middle-income countries (LMICs) [3].

One possible way to address this problem is to design new low-cost low-field strength (LFS) MRI scanners, for which hardware and software are made available as open source [4]. One such initiative is the Open Source Imaging Initiative (OSI^2^) [5]. The development of such an operational LFS system is not possible by simply adapting existing high-field technologies. Instead, new approaches in hardware and software are required [2]. For instance, LFS systems based on Halbach arrays of small permanent magnets is a feasible approach [6–8], with initial prototypes already in use in different sizes and field strengths for various applications, including head examinations [6] and arm examinations [7]. Further LFS systems are based on an iron yoke, for example as a prototype [9, 10] or as a commercial device [11]. To continue this development work, dedicated commitment of numerous scientists worldwide, as well as effective training for incoming young scientists in the field of the currently existing LFS systems are required [12]. In this context, it is worth mentioning the MRI hackathons that now take place regularly [13, 14], where practical skills can be obtained through the development of prototypes. It is particularly effective if scientists from LMICs can be trained on site and thus empowered to contribute new ideas and designs that are tailored for local use [2, 7, 12].

Due to the ease of production and the availability of components, Halbach magnets with field strengths in the range up to $75\text{mT}$ and a field of view (FOV) sufficient to image body parts, like heads, knees or ankles, are primarily designed [6–8, 15]. Hereby, the use of thousands of NdFeB magnets with certain manufacturing tolerances results in a significantly larger inhomogeneity of the magnetic field compared to the idealized simulations. Consequently, it is essential to measure the actual magnetic field in situ and subsequently shim the magnet by adding additional magnets to enhance the field homogeneity in the imaging volume.

The magnetic field measurement is typically carried out with a three-axis positioning system and a commercial Hall sensor, mounted to scan and measure the magnetic field in the center of the magnet [6, 13, 16, 17]. A typical setup has been published by Han et al. in the context of OSI^2^ and is based on a cube-shaped setup [16]. This system is particularly useful for large magnets due to its rigidity and has proven to be reliable in the measurement process.

However, the main disadvantages of this system are the necessity for precise linear drives and size of the mechanical setup, as well as the need for a commercial and precise Hall sensor. If the mechanics are replicated, the material costs are approximately 2000€ at the time of his publication, whereby access to a well-equipped mechanics workshop is necessary. Access to commercial Hall sensors (e.g., from LakeShore Cryotronics, Westerville, USA used in Ref. [8]) is more problematic. Such Hall sensors are precise measuring systems that can easily cost several thousand euros. This expense may be reasonable for the development of a Halbach magnet with a use in diagnostics. In contrast, in the context of training and prototyping, the measurement system should be relatively straightforward and inexpensive, also with the aim of simplifying logistical issues such as customs.

In this work, we have developed a novel system for the measurement of low magnetic fields (e.g., generated by Halbach magnets). This system is based on a self-developed eight-channel Hall sensor (using low-cost automotive chips) and a low-cost robot arm with 5 degrees of freedom (DoF). We have demonstrated the use of this system in shimming a Halbach magnet with $45\text{mT}$ and in the evaluation of a self-designed gradient. Mechanical inaccuracies due to the use of the simple hardware are reliably compensated for by camera-based motion tracking with an ordinary camera, for which only minimal mechanical modifications to the robot are required. The entirety of the software as well as all hardware files are made accessible as open source.

## Methods

### Hall sensor

To identify a suitable compromise between resolution, accuracy, speed, and ease of use, the 3D linear Hall effect sensor ALS31300 (Allegro Microsystems, Manchester, USA) was used. The sensor is available in three different measuring ranges $(\pm 50\text{mT}$, $\pm 100\text{mT}$, $\pm 200\text{mT}$) with the same footprint and software. This allows the appropriate chip to be selected for the desired measuring application without any other hardware changes. The variant ALS31300x-500, with a measuring range of $\pm 50\text{mT}$, was used in the following configuration.

**Fig. 1:**
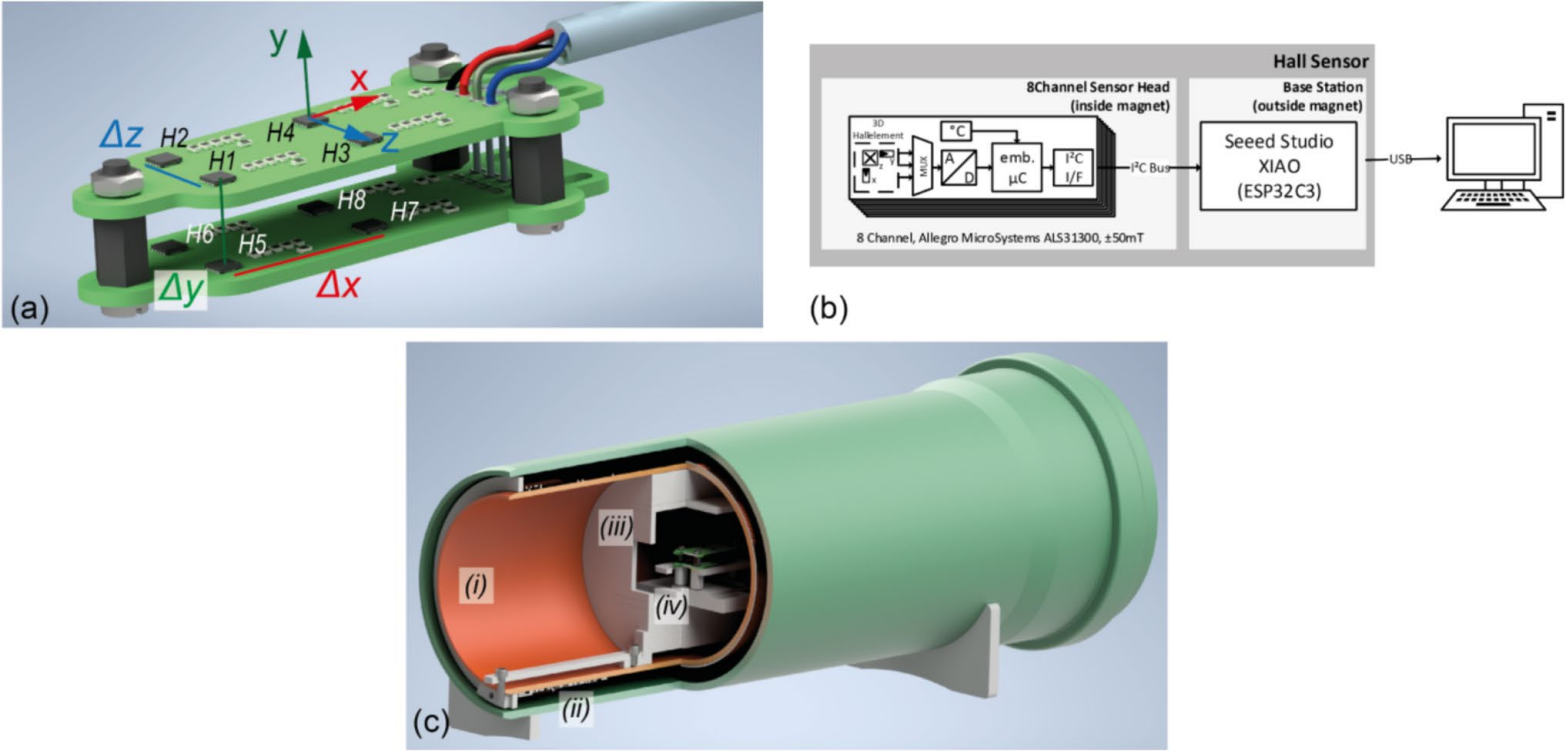
**a **Rendering of the Hall sensor head with 8 Sensors (H1–H8) distributed along 2 planes. **b **Schematic structure of the sensor concept, illustrating the sensor head and a microcontroller-based central control unit. **c **Rendering of the calibration setup, which consists of (i) a double cylinder made of PVC pipe with an inner radius of 151.6 mm, (ii) a two-layer cylindrical coil with 297 layers inside and 27 additional layers at each end, (iii) a positioning slide for the sensor and (iv) the Hall sensor head itself.

The system is divided into two subsystems: a sensor head (see Fig. 1a), on which eight Hall sensors are soldered, and a central control unit with a microcontroller (XIAO ESP32C3, Seeed Technology, Shenzen, China), which controls the Hall sensors, receives the measured values, processes them, and sends them to a computer through USB (schematic in Fig. 1b). The microcontroller software was developed using the Arduino framework (Arduino, www.arduino.cc, Monza, Italy). The sensor head is equipped with eight identical Hall sensors, arranged in a cuboid shape ($\Delta x=20\text{mm}, \Delta y=10\text{mm}, \Delta z=10\text{mm}$), which allows eight measuring points to be measured simultaneously at a single position, thus enabling the mapping of the entire field more quickly.

Each Hall sensor is independently controlled and measures the magnetic field in three orthogonal directions during a single measuring cycle. The integrated analog–digital converter (ADC) has an Effective Number of Bits $\text{ENOB}=10\text{bit}$, with a sensitivity error of $\pm 0.7\%$ ($x$/$z$-axes) and $\pm 0.6\%$ ($y$-axis) over the entire range and a temperature dependence of $0.12\%/\text{K}$. Assuming a proper calibration of the sensor to exclude static errors and static magnetic fields, the resolution accuracy can be significantly enhanced through oversampling. In the developed system, oversampling by a factor of $1000$ was selected, which, in the optimal case of a static error-free system, enables an improvement of the ENOB by $5\text{bits}$ [18]. This results in an ADC-related resolution limit of $3\mu \text{T}$. $1000$ measured values are read in $6.5{\text{s}}$ and sent to the computer as average (a more detailed examination of the averaging and boundary conditions can be found in the Supplementary Information SI). To achieve the maximum sensing performance, a calibration procedure was implemented.

### Calibration of the Hall sensor head

A stable, spatially homogeneous, and precisely known magnetic field is required as a reference for calibrating the Hall sensors. Static electromagnets can be used, as the generated field can be characterized very accurately by Ampère’s law, if the geometry and electrical parameters are known precisely. An easy-to-replicate multi-layer solenoid was designed and used to calibrate the sensor head (see SI, chapter 4), whereby the size of the solenoid is much larger than a single Hall sensor itself. It can, therefore, be assumed that a homogeneous magnetic field is present in the measurement volume of the Hall sensor. The coil is shown in Fig. 1c with the Hall sensor head mounted in the isocenter.

### Robot arm with 5 degrees of freedom

#### Mechanics

**Fig. 2:**
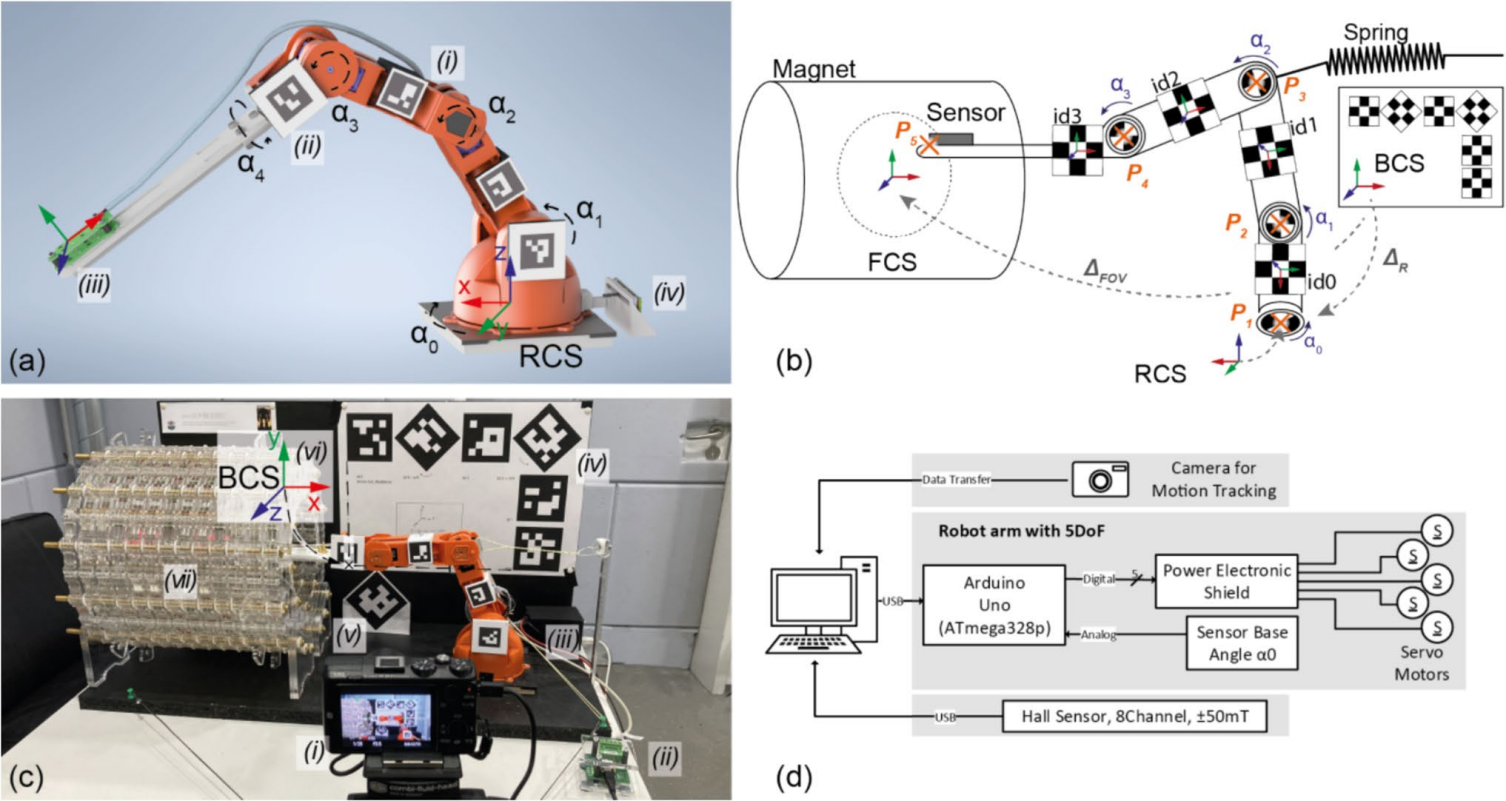
**a **Rendering of the (i) robot with 5DoF, (ii) ArUco marker mechanically fixed to the robot, (iii) mounted Hall sensor and (iv) base angle sensor. **b** Schematic illustration of the coordinate systems of the Base (BCS), the Robot (RCS) and the FOV (FCS), the position of the reference board, the robot markers, the joint angles (α_0_ to α_3_) and the points ***P***_1_ to ***P***_5_ (used in the mathematical description) are presented. **(c) **Picture of the entire setup with (i) the camera, (ii) CPU of the Hall sensor, (iii) Arduino Uno and power electronics shield, (iv) reference board on the back, (v) reference marker at 60 mm towards the camera, (vi) origin of the BCS and (vii) the low field magnet. **d **Control and measurement concept.

A robot arm with 5 DoF is used, with each rotational joint capable of movement up to $180^\circ$ via an integrated servo motor. These robots are also known as manipulators, cobots or redundant robots [19]. In this paper, the open-source robot TinkerKit Braccio from Arduino was used, for which all CAD files are freely available. A rendering is shown in Fig. 2a. However, any robot arm with similar structure can be used, e.g., self-buildable in Ref. [20]. In this work, the commercial version is used and augmented with custom hardware. The $220\text{mm}$-long arm (white in Fig. 2a) ensures that the servo motor is positioned sufficiently far away from the sensor head, whereby the length was estimated using the leakage flux from the motor to the sensor (calculated well below Earth’s magnetic field).

The motor in the shoulder joint (setting ${\alpha }_{1}$) is subjected to the greatest load due to the weight of the whole robot arm. Additional springs are mounted (see Fig. 2b) to reduce the torque requirement from this motor (e.g., $-29.3\%$ torque reduction near the isocenter).

Two global coordinate systems are used: the robot coordinate system (RCS), which has its origin at the base of the robot and is used to describe the kinematics, and the base coordinate system (BCS), which has its origin at the reference board for motion tracking. Both coordinate systems are rotated to each other (see Fig. 2b). The following equation applies to the coordinate transformation for an exemplary matrix ${\varvec{H}}\in {\mathbb{R}}^{3x3}$:

2.1 $${}_{{}}^{RCS} {\varvec{H}} = {}_{{}}^{RCS} {\varvec{R}}_{BCS} {}_{{}}^{BCS} {\varvec{H}} {\text{ with }}{}_{{}}^{RCS} {\varvec{R}}_{BCS} = { }\left( {\begin{array}{*{20}c} { - 1} & 0 & 0 \\ 0 & 0 & 1 \\ 0 & 1 & 0 \\ \end{array} } \right)$$

#### Kinematics

The kinematics of the robot (reduced to a 4DoF problem, with the last axis held constant) is determined by the rotation of the individual axes themselves. The use of homogeneous coordinates in ${\mathbb{R}}^{4x4}$ simplifies the description of the kinematics and, in particular, for the evaluation of the motion tracking, and are, therefore, used in the following analysis (more details in the Appendix). In the kinematics of the robot, each axis is regarded as a separate origin of an individual rotated coordinate system. The origin describes the center of the axis of rotation and is numbered consecutively starting with ${{\varvec{P}}}_{1}$ for the base, continuing with ${{\varvec{P}}}_{2}$ for the next joint and so on. ${{\varvec{P}}}_{5}$ describes the mounting point of the sensor head. Figure 2b illustrates the positions and connections. ${{\varvec{P}}}_{5}$ can be determined by the following affine mapping[21]:

2.2 $${{}^{{\varvec{P}}1}{\varvec{H}}}_{{\varvec{P}}5}={{}^{{\varvec{P}}1}{\varvec{H}}}_{{\varvec{P}}2}{{}^{{\varvec{P}}2}{\varvec{H}}}_{{\varvec{P}}3}{{}^{{\varvec{P}}3}{\varvec{H}}}_{{\varvec{P}}4}{{}^{{\varvec{P}}4}{\varvec{H}}}_{{\varvec{P}}5}$$

where each affine matrix ${}^{{\varvec{P}}{\varvec{i}}}{{\varvec{H}}}_{{\varvec{P}}{\varvec{i}}+1}$ consists of a composition of a rotation ${{\varvec{R}}}_{\boldsymbol{\alpha }{\varvec{i}}}$ and translation ${{\varvec{l}}}_{{\varvec{i}}}$. Once the angles of the joints are known, the position of the sensor head can be calculated with relative ease. Inverse kinematics, however, is an underdetermined problem, as four joint angles must be calculated using only three coordinates [21]. Nonlinear least-squares optimization is used to solve the inverse kinematics [19]:

2.3 $$\tilde{\alpha } = \arg \mathop {\min }\limits_{\alpha } \left( {\frac{1}{2} \left| {\left| {\bf{t}_{\text{goal}} - \bf{t}(\varvec{\upalpha} )} \right|} \right|_{2}^{2} } \right)$$

As a boundary condition, the sensor head should be positioned as horizontally as possible within the FOV to avoid collisions with the magnet bore. Constraining this tilt angle reduces the 3D space, that can be reached by the robot arm, which may result in a discrepancy between the desired and reachable coordinate. A camera-based feedback of the reached position is implemented. The maximum permitted tilt angle (relative to the horizon) was empirically determined as $10^\circ$ in the following experiments. To minimize vibrations of the sensor head, the trajectory through the FOV is planned such that the distance between two adjacent points is minimized. For these roboter types, it has been shown that the higher number of DoF than ultimately required cause difficulties when repeating a trajectory (called repeatability or cyclicity) [19]. An additional experiment was conducted to investigate this error by sequentially approaching the corner points of a cube with a side length of $75{\text{mm}}$ with the center at the isocenter and measuring the magnetic field. This experiment was repeated five time in series.

#### Control of the electronics

The angles to be set are given by the control computer, on which the measurement routine is controlled in a MATLAB (The MathWorks Inc., Natick, USA) script. MATLAB was chosen because of the possible implementation of a feedback control system in the software MATLAB Simulink. A microcontroller board (Arduino Uno) receives the requested angle from the control computer through USB serial interface and translates them into electrical control signals for the servo motors. A power electronics stage between the microcontroller output and the servo motors optimally drives the motors and ensures electrical isolation. This setup is shown schematically in Fig. 2d. The Arduino is programmed in C++, using a self-developed library for the motor control, to enable adjustment of the acceleration of the servo motors. Four of the five servo motors were used to control the position of the sensor head during the measurement. The fifth servo motor enables the sensor head rotation and is set once at the beginning of the measurement and then held in this position. The robot can be powered by a power supply (5V/5A) or battery (e.g., through a voltage regulator from a 12 V lead-acid battery).

### Motion tracking

#### Setup

The combination of inexpensive servo motors and the simple design of the joints results in some mechanical tolerance due to the weight of the robot arm. Vision-based motion tracking is one possible solution to this problem for a variety of robotic systems [22–24] and known as vision-based robot control in case of direct feedback control [25]. The exact absolute position is determined using image recognition-based motion tracking utilizing the free Open Computer Vision (OpenCV) Library [26] (V4.5.5) in conjunction with ArUco (Augmented Reality University of Cordoba) markers [27].

The motion tracking is programmed in Python and the setup consists of three components: the reference board, the markers on the robot, and the camera (shown in Fig. 2c). The reference board comprises seven individual ArUco markers with a size of $80\times80{\text{mm}}$. Six markers are positioned on the back of the setup at known translations and rotations. An additional 7th marker is positioned on an additional support at a distance of $60 \text{mm}$ towards the camera to increase the accuracy of the depth perception of the reference board. These seven markers define the origin of the BCS, which is located on the back of the setup. Four individual ArUco markers measuring $30\times30 \text{mm}$ are attached to the segments of the robot so that the rotation of each joint can be determined by comparing two adjacent markers.

The selection of the used camera is a key factor for the quality of the motion tracking. A selection of tested cameras is listed in the appendix. In this setup, an older digital camera with optical zoom (HX60, Sony, Minato, Tokyo, Japan, f3.5) was chosen. Depending on the camera available, establishing a live connection to the evaluation script is not possible. Therefore, in this work, the video file is analyzed in post-processing. The camera is positioned approximately $70 \text{cm}$ away from the reference board and records the movement at $1920\times1080 \text{px}$ (Full HD) with $25 \text{fps}$. The setup needs to be calibrated to correct for the image distortions by the camera lens [28]. Calibration is performed using a checkerboard pattern with 13 horizontal and 9 vertical elements. $300$ individual images are extracted from a recorded video (length $30 \text{s}$) followed by the automated calculation of the calibration factors (script takes about $1 {\text{min}}$ on a standard office computer). The calculated reprojection error is $0.0685 \text{px}$.

In addition to motion tracking, a self-developed sensor based on a sliding potentiometer has been installed in the system to detect the *y*-Euler angle (which is solely dependent on the base angle ${\alpha }_{0}$). The slider of the potentiometer is moved via a lever mounted to the rotating robot base. A stabilized voltage drops over the potentiometer, allowing the position of the lever to be determined via a voltage measurement $u(x)$. Due to the small angles, a small-angle approximation is employed, resulting in a linear equation (more details in chapter v of the appendix):

2.4 $${\alpha }_{0}={b}_{1}\cdot u\left(x\right)+ {b}_{2}$$

with the two parameters ${b}_{1}=7.6^\circ /V$ and${b}_{2}=-11.7^\circ$, which can be either determined in a calibration measurement, or calculated if the geometry (e.g. distance to the rotation axis) is known precisely. To minimize the tolerances of the sensor due to its mechanical play, the lever of the potentiometer was preloaded using springs.

#### Position determination

OpenCV already has implemented algorithms that can be used to identify ArUco markers within the video file and estimate their position [27]. For each identified marker, the translation and rotation relative to the camera position in the camera coordinate system (CCS, origin in the center of the camera image plane) is calculated.

During the mapping, the robot is given a four-second window to move to the new coordinate and swing out. Thereafter, the position of all markers is evaluated frame by frame, accumulated over 100 frames and then averaged. This averaging of positions serves to enhance the resilience of the system against noise or video errors. In each frame, the translation vector and rotation matrix of the BCS origin in the CCS are determined for all detected reference markers (see chapter vi of appendix for equations). The markers on the robot are detected frame by frame in the CCS according to the same principle and are arithmetically averaged over 100 frames. The robot markers are then coordinate transformed to the BCS, in which the position of the FOV and the RCS are defined by and known from the mechanical setup. To determine the translation and rotation of the sensor head relative to the FOV using the kinematics, it is necessary to determine the actual joint angles. ${\alpha }_{0}$ is known from the additional potentiometer-based sensor and the angles of the joints ${\alpha }_{1}\dots {\alpha }_{3}$ are calculated from the corresponding adjacent markers pair.

#### Reference standard cubic robot using linear drives

**Fig. 3:**
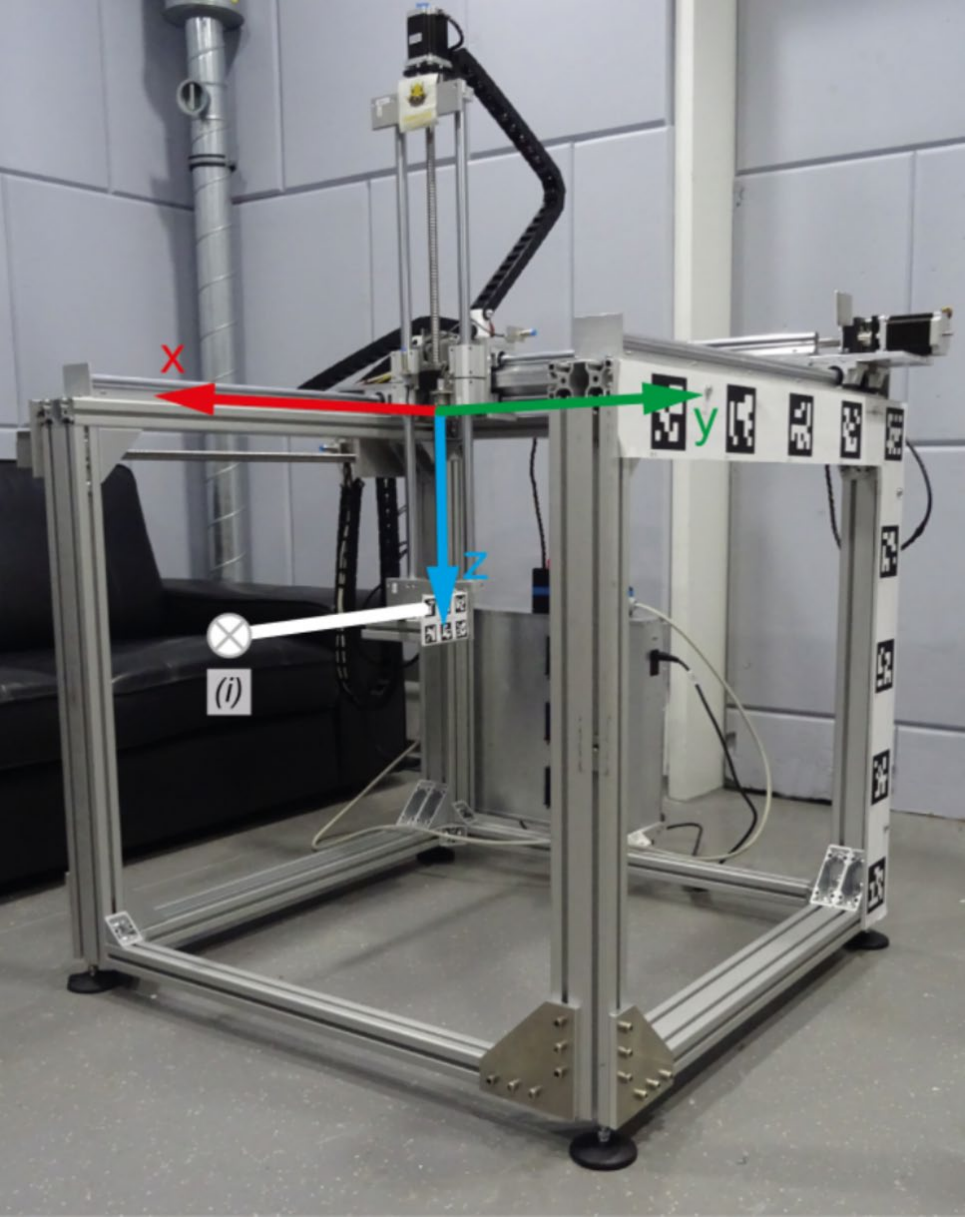
Image of the assembled robot with mounted ArUco markers for precision measurement. It should be noted that a left-handed coordinate system was used in the implementation of this robot. (i) Mounting point of the Hall sensor

To validate the performance of the constructed robotic arm, a standard 3-axis robotic measurement system was replicated according to the design from Han et al.[16]. The robot is shown in Fig. 3 [29]. Due to a different arrangement of the linear drives, the measuring volume could be increased to a volume of $663\times606\times687 \text{mm} (XYZ)$. A commercial Hall sensor (Series 9900 with a 3-axis Hall probe ZOA99-3208, F.W. BELL/MEGGIT, Christchurch, UK) was used, for which an absolute accuracy of $\pm 0.57\%$ (at $50 \text{mT}$, approximately $285\mu T$) and a relative accuracy of $0.035\%$ (at $50 \text{mT}$, approximately $17.5 \mu T$) can be achieved. The low updating rate of the sensor together with an averaging of ten measurements resulted in communication issues between the Hall sensor and the computer, necessitating the termination of the routine. Restarting the Hall sensor initiates a new zeroing, which is reflected in the measured values as a jump of the values. An analysis of the measurement points is only reasonably possible if the data are subsequently processed. For the correction, it is assumed that the same magnetic field strength should be measured at the same point in the FOV in two consecutive measurements. The so calculated offset affects the whole data set, which can yield in an additional error.

The positioning accuracy of the robot was evaluated through motion tracking with OpenCV. Nine ArUco markers (side length $80 \text{mm}$) were affixed to the frame. Two markers (side length $50 \text{mm}$) were mounted on the moving part of the arm. The same camera was used as in the previous section.

### Devices under test

#### Halbach magnet

A Halbach magnet, designed according to O’Reilly et al. [6], was mapped. 536 magnets (NdFeB, $12\times12\times12 \text{mm}^3$ and $1.395 \text{T}\pm 25 \text{mT}$) result in a magnet with length $300 \text{mm}$ and a bore size of $160\text{mm}$. For the purpose of shimming, up to 840 additional magnets (NdFeB, $5\times5\times5 \text{mm}^3$, $1.43T$) can be positioned on the exterior of the Halbach magnet at a radius of $166 \text{mm}$. To compute the additional shimming field, a genetic algorithm according to Refs. [6, 8] was used with considering only the ${B}_{z}$ direction along the ${B}_{0}$ main direction [8] (further information in the SI).

Using the presented measurement setup, a FOV of $75\times75\times75\text{mm}^3$ $(XYZ)$ with a step size of $5 \text{mm}$ was investigated, resulting in a total of 4096 measured points, which corresponds to 512 movements due to the mounted 8 Hall sensors. The entire measurement process took $170 \text{min}$  and was essentially fully autonomous. The FOV is scanned in $yz$-planes consecutively along the $x$-direction. As the $x$ position increases, the force of the spring of the additional bracing, which is intended to mechanically relieve the shoulder motor, decreases. To enhance the precision of the system, the spring was retensioned halfway through the measurement manually. To simplify the shimming process, the measured volume was interpolated to a perfect $5 \text{mm}$ grid.

For completeness, a shimming run was also performed using only the Hall sensor H7, because the eight individual Hall sensors still differ slightly in their calibrated sensitivity due to measurement tolerances during calibration. Thus, using only one sensor for shimming process can be considered as a cross-check. H7 was chosen because this position has the largest number of points around the center of the FOV due to the asymmetric distribution of the measurement points caused by the kinematics.

The same Halbach magnet was measured on a different day using the reference cubic robot and the commercial F.W. BELL Hall sensor, with the possibility of a small temperature-related deviation of ${{\varvec{B}}}_{0}$. A FOV of $100\times100\times100 \text{mm}^3 (XYZ)$ with a step size of $5 \text{mm}$ was measured in a total of 9298 measuring points, which took approx. $38 \text{h}$h and led to splitting the overall measurement into 11 sub-measurements.

#### X-gradient

A $x$-gradient, designed for a spherical FOV of $40 \text{mm}$ using Ref. [30], was mapped with both measuring systems, with the current switched on during the magnetic field measurement of each point (further information in the SI). The current was measured via a shunt ($0.1\%$ tolerance, $\pm 1 \text{ppm}/K$, RUG-Z-R020-0.1-TK1, Isabellenhütte Heusler, Dillenburg, Germany) using a Kelvin measurement and a multimeter (GDM-8255A, GW-Instek, Taipeh City, Taiwan).

Using the presented measurement system, a FOV of $100\times30\times40 \text{mm}^3 (XYZ)$ was mapped with a step size of $10 \text{mm}$, resulting in a total of 288 points (36 movements due to the eightfold sensor head). The measurements were performed with a mean current of $20.403A$. Due to ohmic losses, a convective cooling pause of about $30 \text{s}$  was inserted every 6 movements. The total measurement time was $12 \text{min}$ . The same measurement with the cubic robot took $35 \text{min}$  for 190 points in a spherical FOV with a diameter of $60 \text{mm}$  at a current of $14.5A$ with identical cooling pauses.

As a reference measurement, the gradient was characterized using a clinical 3 T MRI scanner (Prisma, Siemens Healthineers, Erlangen, Germany). For transmit, the body coil was used, while a Flex coil was wrapped around the gradient for receive. The gradient was loaded with a water phantom (0.9 l glass bottle) and it was powered with a current of $0.4 \text{A}.$ To obtain ${B}_{0}$-Fieldmaps, a 3D Gradient-Echo Imaging Sequence was used with the echo times ${T}_{E}=1.9\left|5.16\right|8.42 \text{ms}$ and ${T}_{R}=30 \text{ms}$. A flipangle of $20^\circ$ and an overall isotropic resolution of $2 \text{mm}$ was set. The homogeneity $\Delta {B}_{0}$ was calculated from the phase evolution after the recorded phase was unwrapped [31]. By subtracting a baseline field map, the effect of the turned-on gradient on ${B}_{0}$ itself can be determined.

## Results

### Accuracy of the Hall sensor

**Fig. 4:**
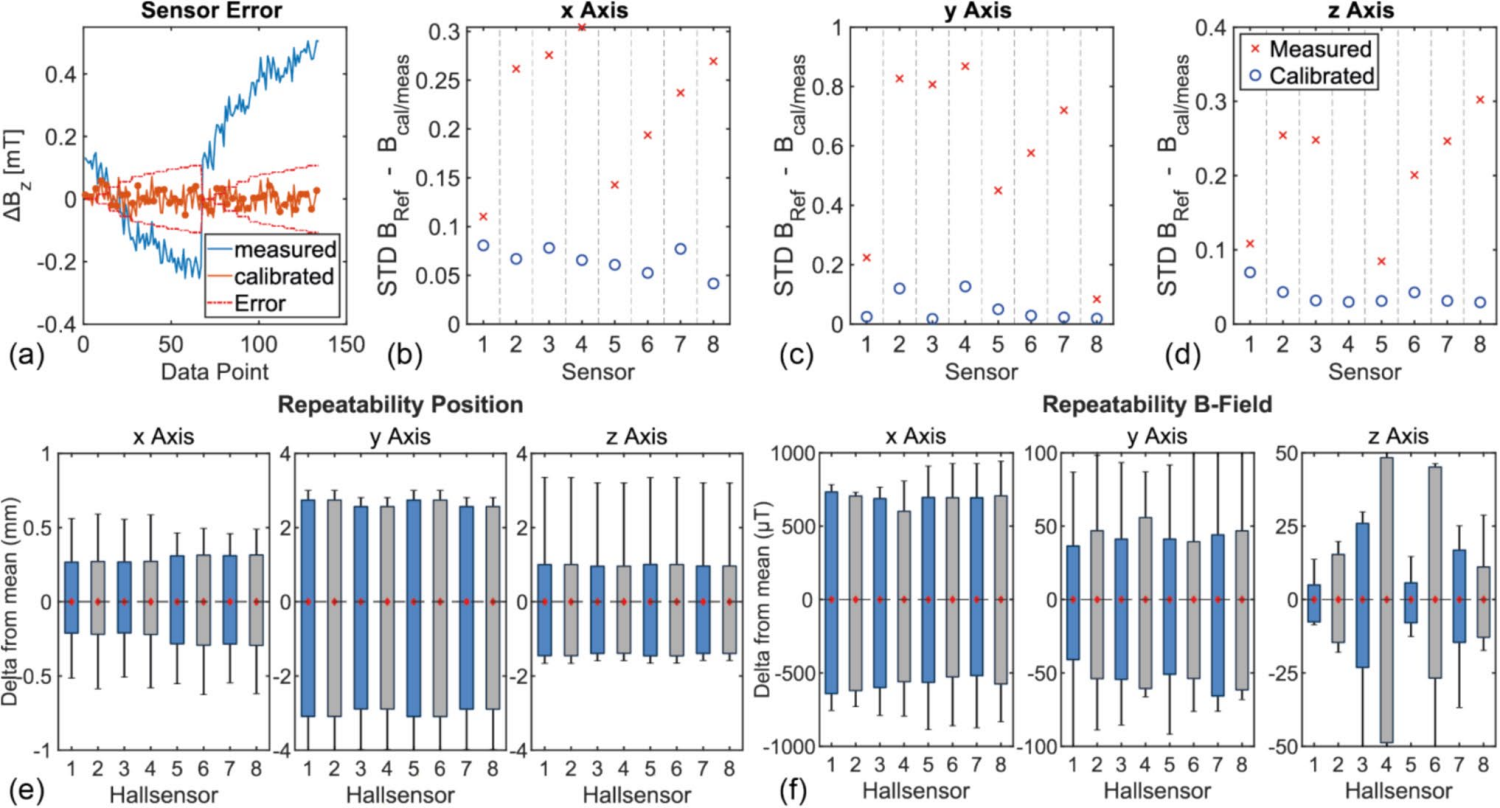
**a **Examples of the error for the z-Axis Hall sensor for the uncorrected and calibrated values for sensor H7. **b–d **Calculated STD of the difference between the applied reference field and the measured value, respective the calibrated value. **e **Repeatability of the moved position as boxplot. The box corresponds to the 25–75% quantile of the data, the whiskers to the extremes, and the red diamond to the arithmetic mean. **f **The measured magnetic field data as boxplot

Figure 4a illustrates a successful calibration example for the exemplary sensor H7 for the $z$-axis. After calibration, a reduction of $34.1-91.2\%$ in the standard deviation (STD) between the measured and applied magnetic fields along the *z*-axis was observed for all eight sensors. An improvement of $23.6\% -85.0\%$ $85.0\%$ was achieved for the *x*-axis. For both axes, the discrepancy between the calibrated value and the applied magnetic field is less than the margin of error of the calibration system. With regard to the *y*-axis, there has been an improvement of $75.6$–$97.9\%$. However, the discrepancy between the measured value and the applied magnetic field remains greater than the margin of error of the calibration system. In addition, some of the eight sensors exhibited a larger calibrated sensitivity error than the manufacturer’s specifications. These errors are likely attributable to the sensors’ reduced sensitivity in the *y*-direction.

### Accuracy of the motion-tracked robot

Figure 4e and f illustrates the result of the repeatability test. Shown is the difference to the averaged value of the experiment. The deviation along the $x$-axis is the smallest and along the $y$-axis (corresponding to the height of the sensor head) it is maximal. The *z*-position of the sensor head is solely dependent on the precision of the base angle sensor. The maximum achievable resolution of the base angle sensor is constrained by the resolution limit of the ADC, which corresponds to $0.03^\circ .$ However, the mechanical play of the potentiometer lever limits the resolution to $0.2^\circ$, which corresponds to a positioning deviation of $\pm 1.5\text{mm}$ for the sensor at the FOV center. This result is confirmed in Fig. 4e. For the magnetic field in Fig. 4f, the measured deviation along the $z$-axis is crucial, since ${{\varvec{B}}}_{0}$ is oriented along this axis and thus the Hall sensors react most sensitively to deviations. The deviation is well below $50\upmu T$ ($<0.11\text{\%}$ of ${{\varvec{B}}}_{0}$). The deviation is larger along the remaining axes.

### Accuracy of the reference standard cubic robot

The deviation between the set position and the actual position was determined in an exemplary volume of $100\times100\times100 \text{mm}^3$ with $\Delta x=4.5\text{mm}, \Delta y=1.2\text{mm}, \Delta z=5.7\text{mm}$ using the motion tracking setup, depending on the direction of movement, which is comparable to the original robot in Ref. [16]. Due to a measuring volume of approximately $5 \text{mm}^3$ of the sensor, this positioning error is acceptable for this volume. Nevertheless, if the positioning error of the robot is analyzed in this manner, this mechanical error can be corrected by an adapted control system.

### Low-field Halbach magnet measurement

**Fig. 5:**
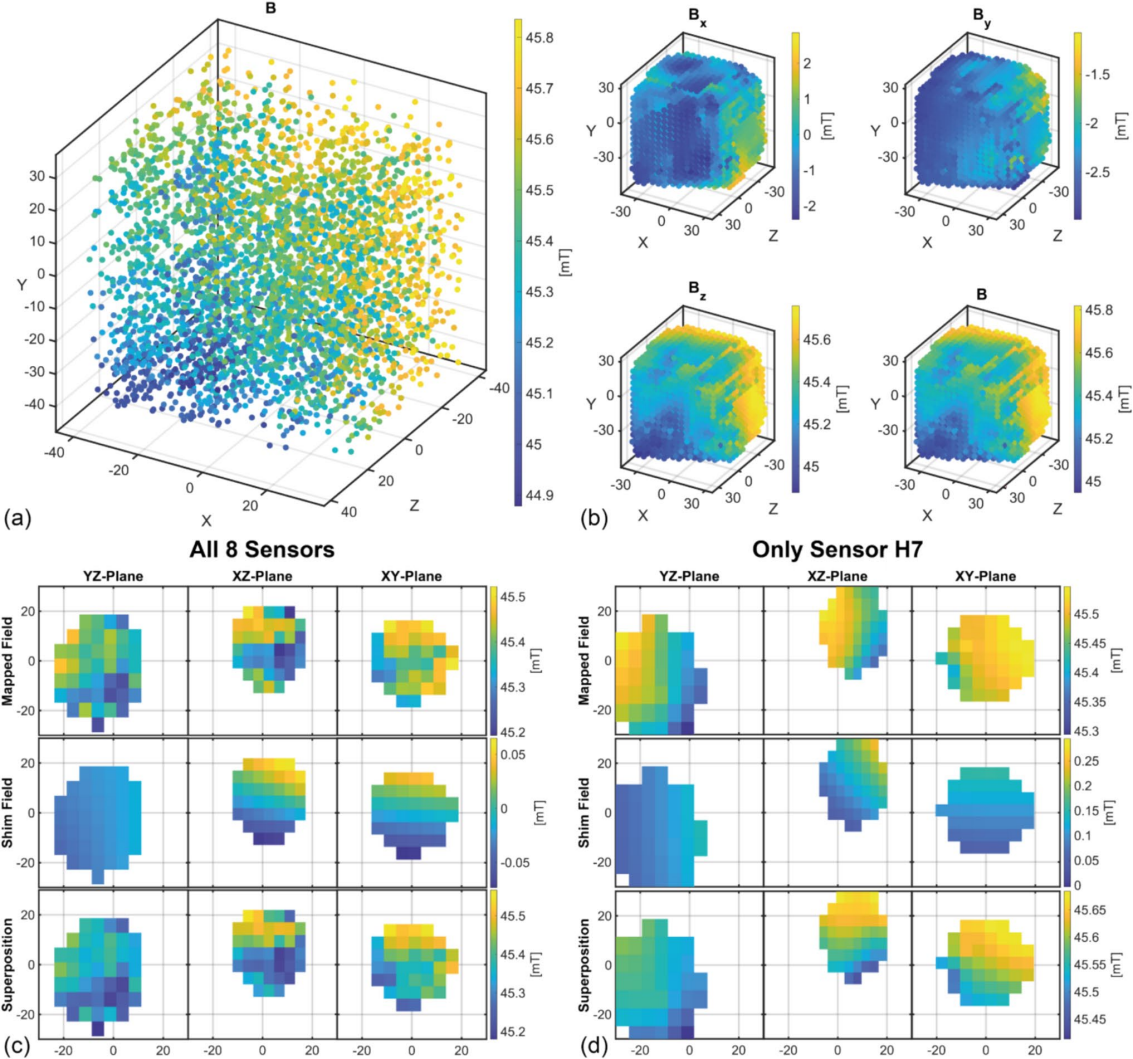
**a **The measured magnitude of the magnetic field is plotted (axes in mm). **b **The components *B*_*x*_*, B*_*y*_, and *B*_*z*_ are plotted, as well as the magnitude of the magnetic field, interpolated to a 5 mm grid (axes in mm). *B*_*x*_*, B*_*y*_, and *B*_*z*_ have been interpolated independently of each other. It should be noted that at this point the measured magnetic field has already been transformed to the BCS coordinate system. **c **Visualization of the calculated shim fields using the interpolated data and the resulting field in the 3 spatial planes. **d **like **c**, but only the sensor H7 was used, which leads to the asymmetrical distribution of points around the center

The measurement setup was tested on the above described Halbach magnet. Due to the orientation of the Halbach magnet with ${B}_{0}$ oriented along the $z$-axis of the BCS, the majority of the field is measured as $B_{z}$ component of the Hall sensors. An analysis of all 4096 measured points indicates that the percentage of $B_{y}$ component in the total measured field by the Hall sensors is less than $1 \%$. Consequently, the error in the calibration of the $y$-axis of the Hall sensors can be neglected. Figure 5 shows the 4096 measured values and the interpolated FOV (6800 points). Due to the kinematics with the selected boundary conditions, not all $y$-positions can be reached with increasing $x$-position, resulting in a slight cut-off of the cubic FOV.

A spherical region of interest with a radius of $20{\text{mm}}$ was set as the volume, which should be shimmed. The mean field is $45.37{\text{mT}}$ with a homogeneity of $12,784{\text{ppm}}$ ($8351{\text{ppm}}$ when interpolated). The possibility of shimming was studied separately for the original data and for the interpolated data. Table 1 shows the shimming results from a single shimming run. With the interpolated data set of all eight sensors, there is a $30.2\%$ improvement in homogeneity. If the data are not interpolated, there is only a $14.1\%$ improvement. It should be noted here that the interpolation acts as a low-pass filter between individual Hall sensors and, therefore, reduces the differences between the individual Hall sensors. This is because adjacent points in the measurement cloud originate from different Hall sensors. This correlation is confirmed by the observation that, for the case of using Hall sensor H7 data exclusively for shimming, a potential improvement in homogeneity of up to $62.5\%$ could be achieved.

**Table 1:** Overview of the different variants of shimming and the results achieved

	Motion Tracked Robot with self-developed Hall sensor			Cubic Robot with commercial Hall sensor	
	All 8 sensors	Interpolated 8 sensors	Just sensor H7	Measured data	Offset cor. data
Before shimming					
* B*_0_ (mT)	45.373	45.377	45.456	42.947	43.068
∆*B*_0_ (ppm)	12784	8351	5870	9314	4642
After shimming					
* B*_0_ (mT)	45.700	45.899	46.444	43.612	43.829
∆*B*_0_ (ppm)	10984	5828	2202	8372	2935
Reduction homogeneity after shimming (1 run) (%)	14.1	30.2	62.5	10.1	36.8
Reduction standard deviation after shimming (%)	25.9	8.9	65.9	14.3	37.9
Amount shimming magnets used	761	727	778	763	784

**Fig. 6:**
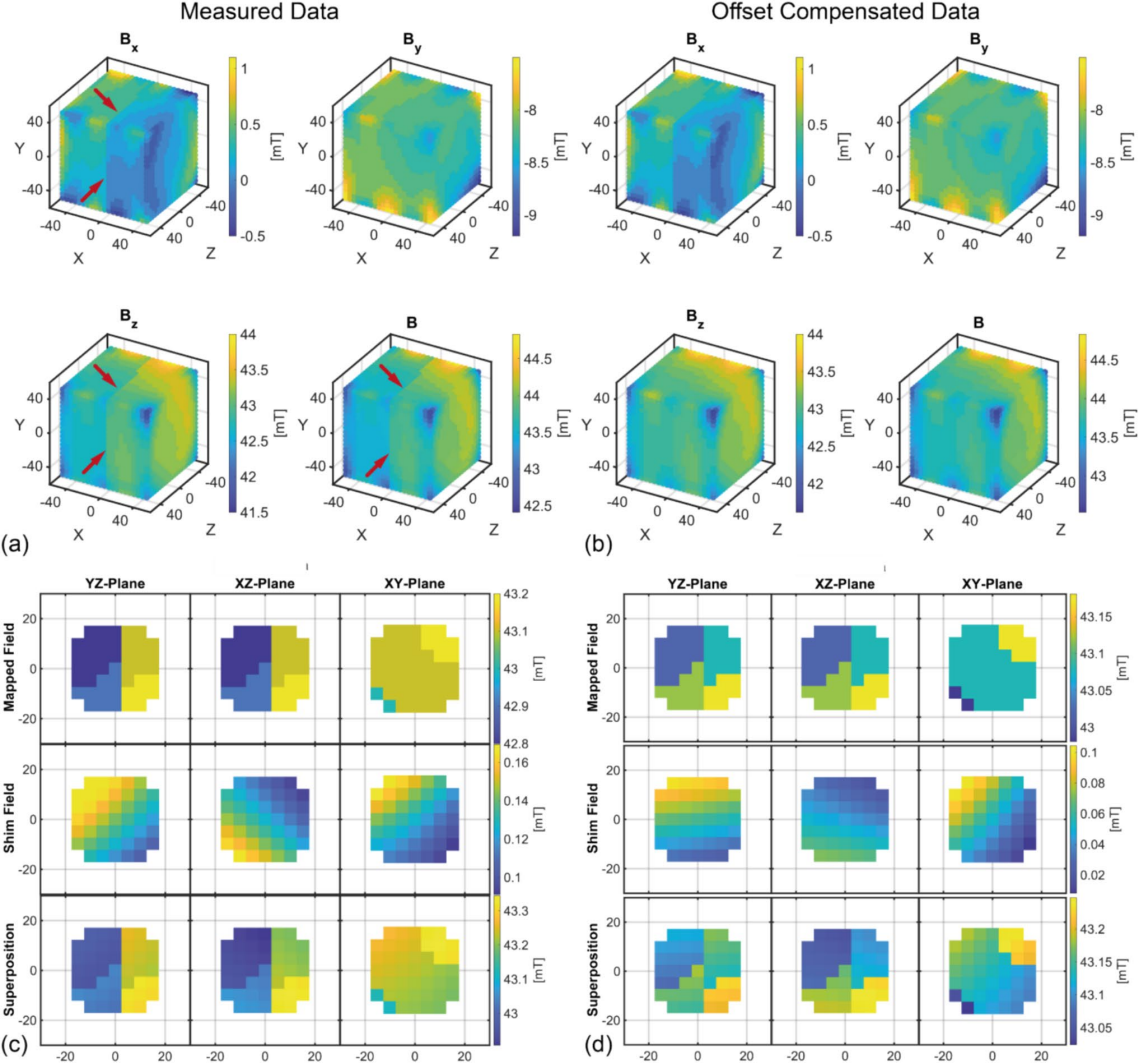
**a **Magnetic field measured with the cubic robot. The red arrows in the data set indicate the jumping offset compensation that occurred after an unintentional restart of the sensor. **b **The offsets in the data set have been corrected. They are almost invisible in the magnitude. **c **The shimming and resulting field calculated from the data set in **a**. It is evident that the jumping offset compensation results in complications. **(d) **The shimming and resulting field calculated from the mapping in **b**. The jumps are still noticeable, but significantly smaller in magnitude. All dimensions in mm

Using the reference cubic robot with the commercial Hall sensor, a lower average field strength of $42.95{\text{mT}}$ (homogeneity of $9314{\text{ppm}}$) is measured for the same FOV size as the first measurement (see Fig. 6), Shimming leads to a reduction of 36.8% if the offset compensated data are used (resp. $10.1\%$ for original data).

### Gradient measurement

**Fig. 7:**
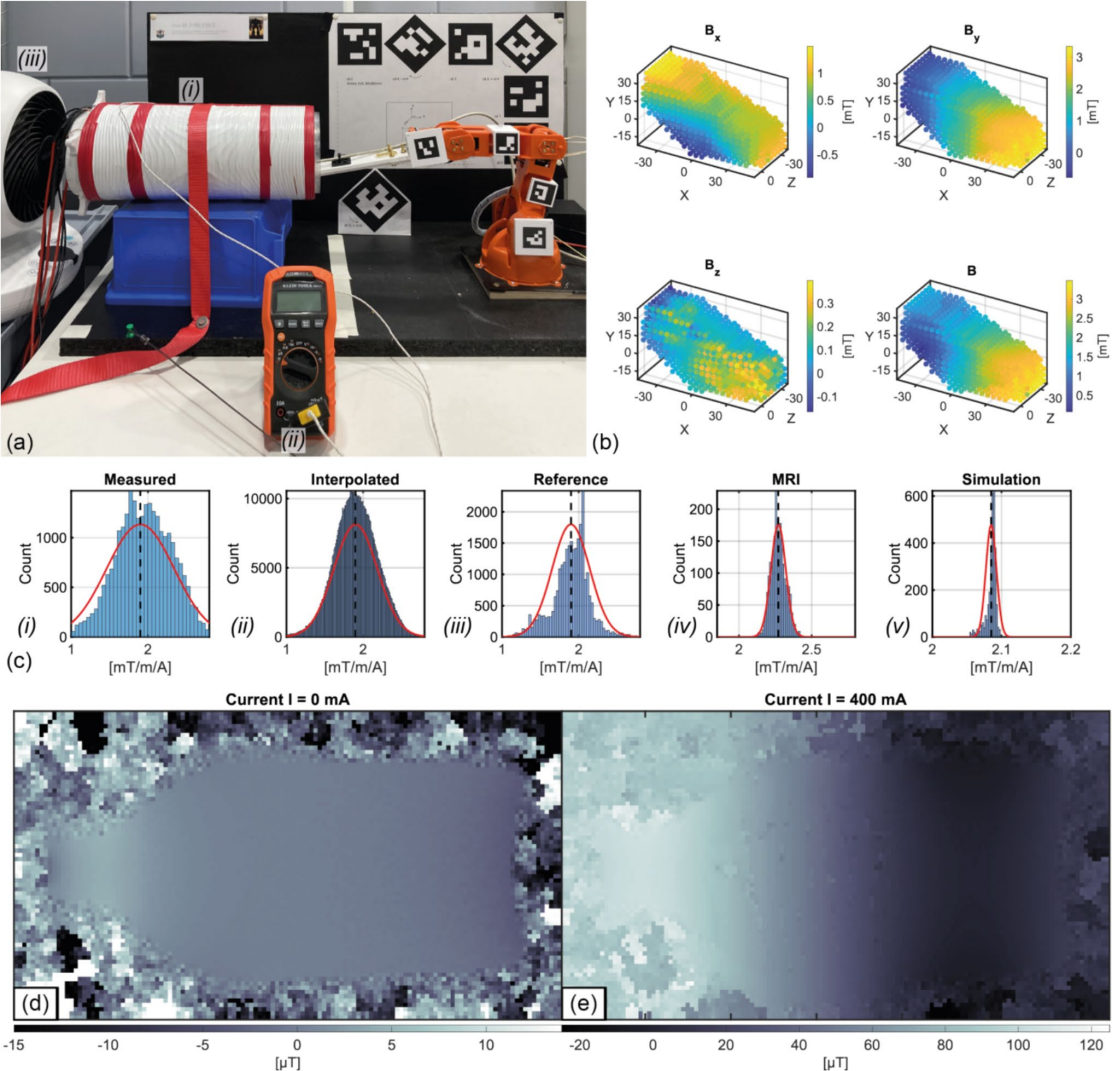
**a **Setup with robot and (i) the gradient, which is fixed in place, (ii) a temperature sensor to monitor the gradient temperature and (iii) a fan to cool the gradient. **b **Plots of the measured field map, acquired using the 5DoF-Robot. **c **The gradient field strength obtained by (i) the 5DoF-Robot, (ii) like (i) but interpolated to a 5mm-grid, (iii) the reference cubic robot with a commercial sensor, (iv) the 3T-MRI measurement and (v) the simulation. **d **B_0_ field map acquired with a 3D GRE sequence, while no current was applied. **e **like **d** but with a current of 400 mA applied

The test gradient coil measurement setup is shown in Fig. 7a. Due to the small measured values (approx. $3{\text{mT}}$ in $y$-direction), the calibration parameters were not used in post-processing. Offset errors are not considered, as only the relative difference between measured values is considered.

The total measurement error is, therefore, dominated by the resolution limit of the motion tracking. The measured points are shown in Fig. 7b (interpolated to an uniform $5{\text{mm}}$ grid). Due to the kinematics of the used robot, not all desired $y$-positions can be reached with increasing $x$-direction, so the reached position is shifted downwards. In post-processing, all measured magnet field values were normalized according to the mean current. The gradient over the entire FOV is calculated between all measurement points as soon as a minimum distance in the $x$-direction of $4{\text{mm}}$ is maintained, resulting in 27,008 data points (722,684 points when interpolated). The standard distribution is shown in Fig. 7c. The calculated gradient strength is shown in Table 2. The same measurement with the cubic robot is shown in Fig. 7c, respective in Table 2. The result of the MRI-based measurement is shown in Fig. 7d and e. The calculated gradient efficiencies from all the measurements are summarized in Table 2. A lower efficiency of the build gradient and a larger variance compared to the simulation is evaluated using the robot-based measurement.

**Table 2:** List of the measured gradient values determined along the *x*-axis from the 4 measurements described above

	Mean value in mT/m/A (deviation from simulation)	Variance *σ* in mT/m/A	FOV size in mm (measured points)
Motion tracked robot with self-developed Hall sensor	1.902 (− 9.6%)	0.440 (0.284 if interpolated)	Cubic, 100×30×40 (288 points)
Cubic robot with commercial Hall sensor	1.899 (− 9.8%)	0.245	Spherical, ∅ 60 (190 points)
MRI scan (3T)	2.271 (+ 8.2%)	0.051	Cubic, 20×20×20 (1000 points)
Simulation	2.085	0.008	Spherical, ∅ 40 (3023 points)

The FOVs differ in the measurements in order to use as many available points as possible for the individual gradient determination

## Discussion

### Hall sensor and positioning system

In general, there are several methods for detecting magnetic fields, such as nuclear magnetic resonance (NMR) [32, 33], tunneling magnetoresistance (TMR) [34], and the Hall effect [35], that could be used for this application as a standalone sensor. NMR-based sensors are characterized by their exceptional precision; however, the necessity for complex and costly electronic components imposes limitations on their application. Integrated TMR sensors, a recent development, possess high sensitivity. To the authors’ knowledge, there are no readily available ICs for 3D measurements in the required field range with integrated signal processing, which is highly desired for an easy-to-build sensor system. The Hall effect, on the other hand, has been intensively investigated, and its integration into commercial electronic chips [35, 36], incorporating three sensing axes and signal processing, renders it a favored option for this work and other low-cost measurement systems [8, 13, 16, 17].

The presented Hall sensor system, comprising eight commercial Hall sensors, demonstrated robustness and reliability during all measurements. As the used main chip is available in different sensing strengths, it is possible to analyze Halbach systems with a field strength up to $200{\text{mT}}$. As high sampling rate is not required for static field measurements, the precision of the sensor can be improved by time averaging the samples; in this work, each measurement consists of 1000 averaged values, which enhances the ENOB to $15bits$. Systematic errors, however, require calibration to utilize this high resolution. In particular, a repeatable calibration proved to be of great importance with regard to ensuring the comparability of the measuring points of the eight individual sensors during the shimming process. A self-developed solenoid as a reference magnetic field was demonstrated to be effective for calibrating the sensors, despite minor imperfections due to manufacturing tolerances.

The positioning system is easy to replicate with various robot arms and is ready for use in a short time. However, low-cost robot arms driven by inexpensive motors need additional position estimation due to limited accuracy and mechanical play. Camera-based motion tracking and ArUco markers attached to the robot proved to be a suitable and reliable solution. Accurately determining the robot base’s rotation angle was determined to be crucial for the overall quality of the position estimation, which was achieved by adding a self-developed sensor using a standard potentiometer.

The repeatability of these robot types is a critical characteristic, given the number of joints involved. The results of the cyclic test demonstrate slight deviations along the $x$- and $z$-axes, which are in the order of magnitude of the sensor itself. The deviation along the *y*-axis is considerably larger, corresponding to the dynamic load of the weight influencing the installed servo motors. The position of the servo motor is controlled by an integrated closed-loop-controller. In this configuration, however, the controller’s tolerance may become too large. The deviation in repeatability is identified through the additional feedback provided by the motion tracking. The ideal magnetic field simulation, when superimposed on these position deviations, yields a range of $Bz \le 20\mu {\text{T}}$, as confirmed by the measurement. For the other axes, the simulated range is considerably smaller. However, the exit angle of the dipole moment is assumed to be ideally perpendicular to the magnet surface in simulation. This is not the case in reality and causes a larger measured deviation. In addition, the Hall sensors are operating at the lower limit of measuring range in the $x$- and $y$-direction, thereby introducing increased noise.

A comparison with cubic robots (e.g., in Refs. [13, 16, 17]) reveals that this robot type appears more complex. However, an analysis of the kinematic equations reveals that the mathematical description of the kinematics is identical in both cases: only in the case of the cubic robot, the joint angles of the 3 DoF are ideally constant at 90°. Based on the authors’ empirical experience, through careful construction, an assembly error of $\pm 0.25^\circ$ of the axes to each other is possible without the need of special tools. Given this error and a FOV with side length $75\times75\times75{\text{mm}}^3$, the resulting positioning distance may be $\left| {\vec{\Delta }} \right| \le 9.8{\text{mm}}$, which is highly dependent on the length of the jib holding the sensor ($1m$ assumed). Adding supplementary feedback has the potential to markedly reduce this error. Therefore, it is reasonable to conclude that the accuracy of the presented system is comparable to that of conventional cubic robots (like the reference cubic robot).

### Measurement of Halbach and gradient

The functionality of the robot and the Hall sensor was demonstrated by mapping a $45{\text{mT}}$ desktop Hallbach magnet, thus enabling the design validation and shimming of the magnet. In comparison to a conventional cubic robot with a commercial Hall sensor, the system presented was able to scan the points approximately six times faster per point, which can be further accelerated by an improved readout software. The determined field strength of the Halbach magnet differs by approximately $2{\text{mT}}$ ($\approx 4.4\%$) between the two Hall sensors (self-developed and commercial sensor), whereby a magnetic field of $48.5{\text{mT}}$ was simulatively expected in the center of the magnet. Based on the calibration of the self-developed sensor and the validation of the calibration with an MRI scanner (see chapter 3 of SI for more information), we assume an incorrect absolute value for the commercial Hall sensor. The reason for this discrepancy remains unclear, as a possible temperature drift due to different measurement days does not fully account for the observed difference.

Both systems showed negligible difference in $x$-gradient measurements. However, the presented system showed greater variance attributed to a larger and asymmetrical FOV. The overall measured gradient efficiency was approximately $9.3\%$ lower than simulations, likely due to mechanical assembly tolerances of the gradient. Reference measurements with a commercial 3 T MRI scanner also showed large deviations, possibly indicating inductive coupling with the installed gradient system or image artifacts due to the copper conductors of the gradient. Despite these issues, the gradient efficiency result differs by only $19\%$ between the MRI reference measurement and the self-developed and calibrated Hall sensor, which could be even less (assuming that the cause of the incorrect MRI evaluation is identified).

### Outlook

In conclusion, the presented system can be utilized in the development of LFS scanners, thereby contributing to both the shimming process of the $B_{0}$ magnetic field and the first evaluation of self-build gradient systems. The developed system’s main advantages include low setup costs (in total approx. 100€), with components like the Hall sensor (approx. 30€), additional robot hardware (approx. 10€), and a low-cost robot (starting from around 50€). In this instance, the components were delivered in a few days. The accessible repository provides a list of potential alternatives to be used in the event of complications. The setup uses accessible digital cameras or smartphones for motion tracking and free software for the motion tracking. The mapping software developed in MATLAB can be migrated to Python in the future, because no special toolboxes were used.

The open-source design and accessible components enable faster assembly and setup times. The system supports fast, self-sufficient, and reliable measurements, beneficial for iterative shimming processes and training new scientists in low-field MRI.

Future improvements in robotic hardware include advanced robotic hardware for more accurate positioning (e.g., by using more powerful motors and gears) and position sensing (e.g., by implementing encoders at the joint axes for feedback). Since it may be difficult to integrate these components in a commercial cheap robot arm, an open-source and redesignable and self-manufacturable robot arm could be used (e.g., in Ref. [20]).

To directly benchmark these robot arms, it would be beneficial to design an improved and simplified experiment for absolute spatial accuracy (as in Ref. [16]). However, a direct transfer is not feasible due to kinematic constraints, which inhibit a perpendicular projection of the laser beam onto the paper. One potential solution involves mounting the laser on a gimbal to compensate for the tilt angle of the last robot segment. The Hall sensor data quality could be significantly improved by implementing an enhanced calibration process using more reliable setups (e.g., in Ref. [37]). This sensor architecture is usable up to $200mT$. In case higher magnetic fields should be mapped (e.g., in Ref. [38]), a redesign of the sensor head is necessary: for up to $3T$ 1-axis Hall sensors with analog outputs (e.g., CYSJ106C, Chenyang Technologies, Fising, Germany) are available, whereby 3 mounted sensors could be digitalized using one I^2^C-capable ADC (e.g., 4 Channel TLA2024, Texas Instruments, Dallas, USA).

Components of the system, like motion tracking, can be reused for other applications, such as recording patient movements during examinations, demonstrating the system’s versatility and educational value.

## Electronic supplementary material

Below is the link to the electronic supplementary material.

Supplementary file1 (DOCX 4732 KB)

Supplementary file2 (TIF 13608 KB)

Supplementary file3 (TIF 5149 KB)

Supplementary file4 (TIF 8060 KB)

Supplementary file5 (TIF 7693 KB)

Supplementary file6 (TIF 4539 KB)

## Figures and Tables

**Fig. 8:**
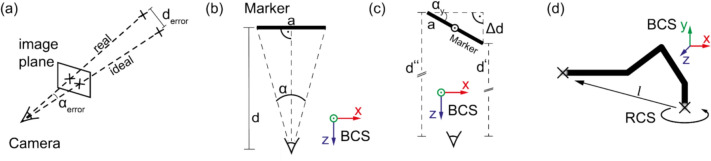
Sketches to illustrate the geometry used to determine the motion tracking accuracy: **a** illustration of the reprojection error of the camera calibration, **b** determination of the error in the $xy$-plane, **c** determination of the error of the Euler angle around the $y$-axis, **d** impact on an inaccurate $y$-Euler angle on the position estimation along the $z$-axis

**Fig. 9:**
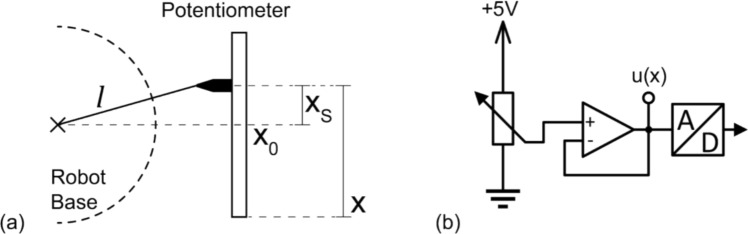
**a** Schematic illustration of the geometric dimensions of the robot base and the potentiometer with the lever in between. **b** Electrical equivalent circuit diagram of the potentiometer, the internal ADC, and an impedance converter in between

**Fig. 10:**
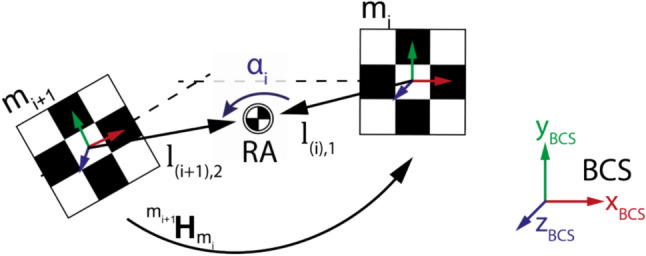
Schematic representation of the mechanical connection between a joint and the neighboring two robot markers

## Data Availability

The source code of the software (robot control, motion tracking, embedded code sensor, evaluation of magnetic field mapping, simulation of magnetic fields) as well as the hardware files (PCB files, calibration cylinder, CAD robot) to reproduce or extend this work are available under https://github.com/ppolGTMonster/MiniBumblebee_LowFieldMapping.

## Appendix

### i. Homogenous coordinates

The following equation applies to the transformation of a $3{\text{x}}1$-vector ${\varvec{t}}$ (Eq. (10.1)) or a $3{\text{x}}3$-matrix ${\varvec{R}}$ (Eq. (10.2)) into homogenous coordinates:

10.1 $${\varvec{A}}_{HC} = \left( {\begin{array}{*{20}c} \bf{0}_{3x3} & {\varvec{t}} \\ \bf{0}^{T} & 1 \\ \end{array} } \right) \in {\mathbb{R}}^{4x4}$$

10.2 $${\varvec{A}}_{HC} = \left( {\begin{array}{*{20}c} {\varvec{R}} & \bf{0} \\ \bf{0}^{T} & 1 \\ \end{array} } \right) \in {\mathbb{R}}^{4x4}$$

with the null vector $\bf{0} \in {\mathbb{R}}^{3}$ and the null matrix $\bf{0}_{3x3} \in {\mathbb{R}}^{3x3}$.

### Affine mapping to determine Point P5

Rotation matrices and translations are required to determine the position and rotation of the Hall sensor from the angles of the joints. The rotations are calculated as follows (rotation $\bf{R}_{\alpha 0}$ is a rotation around the $z$-axis; $\bf{R}_{\alpha 1}$, $\bf{R}_{\alpha 2}$, $\bf{R}_{\alpha 3}$ are rotations around the $y$-axis):

$${\varvec{R}}_{{{\varvec{\alpha}}0}} = \left( {\begin{array}{*{20}c} {\cos \alpha_{0} } & { - \sin \alpha_{0} } & 0 & 0 \\ {\sin \alpha_{0} } & {\cos \alpha_{0} } & 0 & 0 \\ 0 & 0 & 1 & 0 \\ 0 & 0 & 0 & 1 \\ \end{array} } \right)$$

10.3 $${\varvec{R}}_{{\varvec{\alpha i}}} = \left( {\begin{array}{*{20}c} {\cos \alpha_{i} } & 0 & {\sin \alpha_{i} } & 0 \\ 0 & 1 & 0 & 0 \\ { - \sin \alpha_{i} } & 0 & {\cos \alpha_{i} } & 0 \\ 0 & 0 & 0 & 1 \\ \end{array} } \right) \,\,for\,\, i = 1,2,3$$

The translations are defined by the structure of the robot used, where by definition at an angle of $0^\circ$, the extension is along the $z$-axis in the RCS:

10.4 $${\varvec{l}}_{{\varvec{i}}} = \left( {\begin{array}{*{20}c} 0 & 0 & 0 & 0 \\ 0 & 0 & 0 & 0 \\ 0 & 0 & 0 & {l_{i} } \\ 0 & 0 & 0 & 1 \\ \end{array} } \right)\,\, for\,\, i = 0,1,2,3$$

with $$l_{0} = 71.6\,\,{\rm mm}$$, $$l_{1} = 124.4\,\,{\rm mm}$$,$$l_{2} = 124.6\,\,{\rm mm}$$, $$l_{3} = 280.7\,\,{\rm mm}$$.

### Camera selection for motion tracking

The selection of camera is determining the quality and accuracy of motion tracking. A multitude of camera types exist, including webcams, photographic cameras, smartphones, and industrial cameras, each of which possesses inherent limitations. Notable differences emerge in terms of resolution, integrated optical lenses, and video data access. Table 3 provides an overview of the cameras evaluated in the experimental setup. The implemented motion tracking is designed to accommodate all available camera types, with the tracked positions and magnetic field data being merged in post-processing. During the magnet mapping, technical limits to the recording duration may be encountered, which are addressed by a short pause in recording, followed by its subsequent restart:

- The maximum recording time for inexpensive photographic cameras is typically technically limited to 30 min (due to customs laws in the European Union).
- Digital single-lens reflex (DSLR) cameras may overheat during recording, necessitating a cooling period. However, the lenses employed ensure that the captured recording exhibits exceptional quality.
- The data memory may limit the maximum size of the video file.
- Battery life can also be an limiting factor

**Table 3:** A comparison of the cameras tested in the setup. The cameras were calibrated and evaluated in the same way. The camera Sony HX60 was used in the experiment

Type	Camera used	Resolution	Calculated reprojection error (scaled to HD)	Deviation to camera used in experiment	Direct accessibility in OpenCV
Digital single-lens reflex camera	Sony Alpha 7 III with lens Sigma FE24-150 f/4 G OSS (2021–3500€)	4K (3840 × 2160px)	0.0260 px	− 62.0%	Partially through extra libraries (e.g., libgphoto2 with full control)
Smartphone	Apple iPhone SE (2020-450€)	4K (3840 × 2160px)	0.0405 px	− 40.8%	No (except using third party software like virtual webcams)
Digital camera with optical zoom	Sony HX60 (2015–260€)	FullHD (1920 × 1080px)	0.0684 px	0%	Partially through extra libraries (e.g., libgphoto2 with limited control)
Smartphone	Apple iPhone SE (2020, 450€)	FullHD (1920 × 1080px)	0.0776 px	+ 13.3%	No (except using third party software like virtual webcams)
Webcam (digital camera with digital zoom)	Logitech C920s Autozoom (2023–100€)	FullHD (1920 × 1080px)	0.0873 px	+ 27.5%	Yes
	Logitech C920s Manual Zoom (2023–100€)	FullHD (1920 × 1080px)	0.0892 px	+ 30.3%	Yes
Webcam	Renkforce RF-WC-150 (2024–20€)	FullHD (1920 × 1080px)	0.2526 px	+ 268.9%	Yes

In best case, a high-quality (4 K or more) webcam or industrial camera is available, allowing simple live access to the video material. In this study, an older photographic FullHD-camera (Sony HX60) was used to illustrate the functionality of the presented setup with a camera type that is readily available.

### Accuracy of the motion tracking

The translation of the robot markers relative to the BCS can be determined with great precision using OpenCV, if the camera is properly calibrated. A reprojection error is calculated from the calibration algorithm with $e = 6.8475 \cdot 10^{ - 2} {\text{px}}$. The camera utilized (DSC-HX60, Sony, Minato, Tokyo, Japan) has a field of view angle of ${\text{FOV}}_{{{\text{angle}}}} = 84^\circ$ at the lowest focal length, with a resolution of $w_{px} \times h_{px} = 1920 \times 1080px$. The camera is located at a distance of $681{\text{mm}}$ from the reference board and $481{\text{mm}}$ from the robot base.

For the sake of simplicity, it is assumed that the angle of view is distributed uniformly across the width of the image. Consequently, the so-called pixel angle along the $x$-axis can be expressed as:

10.5 $$\alpha_{px,w} \approx \frac{{{\text{FOV}}_{{{\text{angle}}}} }}{{w_{px} }} = 0.0438 ^\circ {\text{px}}^{ - 1}$$

The calibration-related angle error is calculated to be (see Fig. 8a):

10.6 $$\alpha_{\text{error}} = \alpha_{px,w} \cdot e = 2.996 \cdot 10^{-3^{\circ}}$$

using simple trigonometry with (see Fig. 8a):

10.7 $$\tan \alpha_{{{\text{error}}}} = \frac{{d_{{{\text{error}}}} }}{d}$$

The error of the position estimation along the $x$-axis results in $35.6\mu m$ for the reference board, resp. $24.1\mu {\text{m}}$ for the robot markers and can be, therefore, neglected, because it is smaller than the measurement volume of the hall sensor (according to the data sheet, an area with a side length of $150\mu m$ is spanned along the $x$- and $z$-axes). The camera’s angle of view along the $y$-axis is smaller, yet the number of pixels is also smaller. It can, therefore, be assumed that the error is of a similar size.

Significantly greater difficulties arise for the position estimation along the *z*-axis, which is defined by the Euler angle around the $y$-axis. This is due to the fact that small angle changes only cause minor image changes due to the projection onto the 2D image plane of the camera parallel to the $xy$-plane. To estimate this error, a simplified model is used: an ArUco marker with side length $a$ at a distance $d$ from the ideal camera corresponds to a viewing angle in the $xy$-plane of (see Fig. 8b):

10.8 $$\tan \frac{\alpha }{2} = \frac{a}{2d}$$

If the marker is rotated around the $y$-axis, the distance to the camera can be calculated for both edges in the $xy$-plane (see Fig. 8c):

10.9 $$d^{\prime} = \frac{a}{{2\tan \frac{\alpha ^{\prime}}{2}}}$$

10.10 $$d^{\prime\prime} = \frac{a}{{2\tan \frac{\alpha ^{\prime\prime}}{2}}}$$

10.11 $$\Delta d = d^{\prime \prime} - d^{\prime}$$

The following relationship applies in the $xz$-plane (using (10.11)):

10.12 $$\sin \alpha_{y} = \frac{\Delta d}{a} = \frac{1}{{2\tan \frac{{\alpha^{\prime\prime}}}{2}}} - \frac{1}{{2\tan \frac{{\alpha^{\prime}}}{2}}}$$

If the marker is not rotated, the maximum possible deviation for the Euler angle $a_{y}$ can be calculated using (10.12) and the angle error $\alpha_{error}$ from (10.6):

10.13 $$\Delta \alpha_{y} = \pm \left| {\sin^{ - 1} \left( {\frac{1}{{2\tan \frac{{\alpha + \alpha_{{{\text{error}}}} }}{2}}} - \frac{1}{{2\tan \frac{{\alpha - \alpha_{{{\text{error}}}} }}{2}}}} \right)} \right|$$

For a robot marker ($d = 481{\rm mm}$, $a = 30{\rm mm}$), an angle of $\alpha = 3.57^\circ$ results in a range of the calculated Euler angle around the $y$-axis of $\Delta \alpha_{y} = \pm 1.54^{^\circ }$. One possible point in the FOV illustrates the resulting error of the position estimation. If the point to be approached is the center of the FOV, which is $\Delta x = 430 {\text{mm}}$ away from the robot base ($z$-coordinate of both points at $z = 200{\rm mm}$), the $z$-error can be calculated using simple trigonometry (see Fig. 8d). Let the translation between the robot base and the Hall sensor be the vector ${\varvec{l}} = \left( {430mm,y,0} \right)^{T}$ and the rotation matrix around the $y$-axis ${\varvec{R}}_{{\varvec{y}}} \left( {\alpha_{y} } \right)$, which is a unit matrix for $\alpha_{y} = 0^\circ$. The measurement error of the angle results in a different rotation matrix $R_{y} \left( {\Delta \alpha_{y} = \alpha_{y} } \right)$, so that the calculated position for both rotations differ by the vector:

10.14 $$\Delta \bf{l} = \left( {\bf{R}_{y} \left( {\Delta \alpha_{y} } \right) - \bf{R}_{y} \left( {0^{^\circ } } \right)} \right) \cdot \bf{l}$$

where, in this specific example, the error along the $z$-axis becomes:

10.15 $$\Delta z{\mkern 1mu} = {\mkern 1mu} \pm {\text{sin}}\Delta \alpha _{y} \cdot \Delta x$$

Using (10.13), an error on the $z$-axis can be calculated with $\Delta z = \pm 11.6{\text{mm}}$.

Rotations around the $z$-axis occur within the image plane, so that minor changes can be measured precisely. In contrast, rotations around the $x$-axis do not occur within the setup system, as the last joint of the 5 joints is held static.

### Accuracy of the additional sensor to measure the base angle

The movable slide of the sliding potentiometer is connected to the rotating base of the robot via a lever (see Fig. 9a). A stabilized $U_{cc} = 5V$ drops via the potentiometer, and the middle voltage is digitized with the ADC of the Arduino. An impedance converter in between increases the isolation and therefore accuracy of the voltage divider (see Fig. 9b). The absolute position $x$ of the lever can be calculated using the voltage measurement:

10.16 $$x = \frac{u\left( x \right)}{k}$$

with the sensitivity:

10.17 $$k = \frac{{U_{cc} }}{x^{\prime}}$$

and the effective maximum electrical length $x^{\prime}$ of the potentiometer, where the following holds:

10.18 $$u\left( {x = x^{\prime}} \right) = U_{cc}$$

The relative position of the lever can be calculated with:

10.19 $$x_{s} = x - x_{0}$$

where $x_{0}$ is the standby position ($\alpha_{y} = 0^\circ$). The following relationship applies to the angle of rotation using a small-angle approximation:

10.20 $$\alpha = \sin^{ - 1} \frac{{x_{s} }}{l} \approx \frac{180^\circ }{\pi } \cdot \frac{{x_{s} }}{l}$$

Using (10.16)–(10.19), the following linear relationship can be obtained:

10.21 $$\alpha \left[^\circ \right] = b_{1} \cdot u\left( x \right) + b_{2}$$

with the two parameters:

10.22 $$b_{1} = \frac{180^\circ }{{\pi \cdot k \cdot l}}$$

10.23 $$b_{2} = \frac{180^\circ }{{\pi \cdot l}} \cdot x_{0}$$

The measurement of the length $l$, the effective maximum electrical length $x^{\prime}$ (and thus $k$), and the exact center position $x_{0}$ is challenging by hand using a caliper etc.

Therefore, a calibration measurement was implemented, where a self-leveling rotation laser (GLL 3–80, Bosch, Stuttgart, Germany) was mounted on the basis of the robot. The geometric movement of the laser projection was then measured on a screen at a distance of $595{\rm mm}$ (measurement error of laser movement in total approx. $0.05^\circ$ due to the laser beam width). The measured data were utilized for a linear regression analysis of (10.21) resulting in $b_{1} = 7.6^\circ /V$ and $b_{2} = - 11.7^\circ$, which matches with the equations in (10.22) and (10.23) and manually measured parameters $l$, $x^{\prime}$ and $x_{0}$. The ADC built in the Arduino (ATmega328 microcontroller) has ${\text{ENOB}} = 9.8\;{\text{bits}}$, which, when combined with the used reference voltage of the ADC $(U_{{{\text{ref}}}} = 3.745\;{\text{V}})$, yields to a resolution limit of $\Delta U = 4.2\;{\text{mV}}$. Using (10.16) results in a minimum resolution of $\Delta \alpha_{{{\text{min}}}} = 0.03^{^\circ }$. However, the overall resolution of the sensor is mainly influenced by the mechanical play of the potentiometer itself. Consequently, the potentiometer was preloaded with a spring in the negative direction to facilitate the slider. This results in a maximum error of $\Delta \alpha_{{{\text{sum}}}} = \pm 0.2^{^\circ }$, which was determined from the calibration data. Using (10.15) results in an error of $\Delta z = \pm 1.5\;{\text{mm}}$.

### Further Information on the mathematics of the motion tracking

#### Calculating the marker position and coordinate transformation of the reference board

The initial stage of motion tracking is the accurate identification of markers on the reference board, which enables the determination of the position and rotation of the BCS in the CCS. To achieve this, the position of the BCS origin is calculated for each detected marker in each frame:

10.24 $${}_{{}}^{CCS} {\varvec{H}}_{BCS}^{{}} = {}_{{}}^{CCS} {\varvec{H}}_{Mi}^{{}} \cdot \left( {{}_{{}}^{BCS} {\varvec{H}}_{Mi}^{{}} } \right)^{ - 1}$$

where ${}_{{}}^{CCS} {\varvec{H}}_{Mi}^{{}}$ is the determined homogeneous coordinates of marker $i$ to the CCS and ${}_{{}}^{BCS} {\varvec{H}}_{Mi}^{{}}$ is the known translation and rotation of this marker in the BCS. Subsequently, the translation vector and the rotation matrix are derived from this homogeneous coordinate and averaged. The translation is simply the arithmetic mean in ${\mathbb{R}}^{3}$:

10.25 $${}_{{}}^{CCS} {\varvec{t}}_{BCS}^{{}} = \frac{1}{n}\mathop \sum \limits_{i = 1}^{n} {}_{{}}^{CCS} {\varvec{t}}_{BCS,i}^{{}} { } \in {\mathbb{R}}^{3}$$

It is not meaningful to simply average rotation matrices arithmetically. An alternative approach would be to average the Euler angles determined from the rotation matrix. However, there are critical angles that lead to a singularity of the system (so-called gimbal lock). To avoid this issue, the rotation matrices are converted into quaternions. Using the Euler–Rodrigues formula, a transformation of matrices in ${\mathbb{R}}^{3x3}$ into the set of quaternions in ${\mathbb{H}}$ is possible. The transformation is done according to the following equations:

10.26 $$q = q_{0} + i \cdot q_{1} + j \cdot q_{2} + k \cdot q_{3} \in {\mathbb{H}}$$

10.27 $$R = \left( {\begin{array}{*{20}c} {1 - 2\left( {q_{2}^{2} - q_{3}^{2} } \right)} & { - 2q_{0} q_{3} + 2q_{1} q_{2} } & {2q_{0} q_{2} + 2q_{1} q_{3} } \\ {2q_{0} q_{3} + 2q_{1} q_{2} } & {1 - 2\left( {q_{1}^{2} - q_{3}^{2} } \right)} & { - 2q_{0} q_{1} + 2q_{2} q_{3} } \\ { - 2q_{0} q_{2} + 2q_{1} q_{3} } & {2q_{0} q_{1} + 2q_{2} q_{3} } & {1 - 2\left( {q_{1}^{2} - q_{2}^{2} } \right)} \\ \end{array} } \right) \in {\mathbb{R}}^{3x3}$$

The obtained quaternions are then averaged arithmetically:

10.28 $${}_{{}}^{CCS} q_{BCS}^{{}} = \frac{1}{n}\mathop \sum \limits_{i = 1}^{n} {}_{{}}^{CCS} q_{BCS,i}^{{}} \in {\mathbb{H}}$$

where ${}_{{}}^{CCS} q_{BCS,i}^{{}}$ is the converted rotation matrix of one detected marker $i$.

#### Determination of the robot’s joint angles

The following concatenated mapping can be utilized to ascertain the transformation from a marker $m_{i}$ to its adjacent marker $m_{i + 1}$, considering the already determined positions and rotations of the two markers (where RA denotes the rotation axis of the joint lying between the markers). In general, the position of the marker $m_{i + 1}$ can be written as (refer to Fig. 10):

10.29 $${}_{{}}^{BCS} {\varvec{H}}_{{m_{i + 1} }}^{{}} = {}_{{}}^{BCS} {\varvec{H}}_{{m_{i} }}^{{}} {}_{{}}^{{m_{i} }} {\varvec{H}}_{RA}^{{}} {}_{{}}^{RA} {\varvec{H}}_{{m_{i + 1} }}^{{}} {}_{{}}^{{m_{i + 1} }} {\varvec{H}}_{{m_{i} }}^{{}} {}_{{}}^{{m_{i} }} {\varvec{H}}_{RA}^{{}} {}_{{}}^{RA} {\varvec{H}}_{{m_{i + 1} }}^{{}}$$

The joint angle is part of the matrix ${}_{{}}^{{m_{i + 1} }} {\varvec{H}}_{{m_{i} }}^{{}}$ which can be calculated by:

10.30 $${}_{{}}^{{m_{i + 1} }} {\varvec{H}}_{{m_{i} }}^{{}} = {}_{{}}^{{m_{i + 1} }} {\varvec{H}}_{RA}^{{}} {}_{{}}^{RA} {\varvec{H}}_{{m_{i} }}^{{}} {}_{{}}^{{m_{i} }} {\varvec{H}}_{BCS}^{{}} {}_{{}}^{BCS} {\varvec{H}}_{{m_{i + 1} }}^{{}} {}_{{}}^{{m_{i + 1} }} {\varvec{H}}_{RA}^{{}} {}_{{}}^{RA} {\varvec{H}}_{{m_{i} }}^{{}}$$

where the homogeneous coordinate between one marker and the rotation axis can be calculated as:

10.31 $${}_{{}}^{{m_{i} }} {\varvec{H}}_{RA}^{{}} = \left( {\begin{array}{*{20}c} {\bf{1}_{3x3} } & {{\varvec{l}}_{i,1} } \\ {\bf{0}^{T} } & 1 \\ \end{array} } \right)$$

10.32 $${}_{{}}^{{m_{i + 1} }} {\varvec{H}}_{RA}^{{}} = \left( {\begin{array}{*{20}c} {\bf{1}_{3x3} } & {{\varvec{l}}_{i,2} } \\ {\bf{0}^{T} } & 1 \\ \end{array} } \right)$$

with the identity matrix $\bf{1}_{3x3} \in {\mathbb{R}}^{3x3}$ and the null vector $\bf{0} \in {\mathbb{R}}^{3}$.

The vector between the marker position and the rotation axis can be easily measured in the CAD model. For the robot utilized in this study, the lengths are described in Table 4. The Euler angle around the $z$-axis is identical to the wanted joint angle.

**Table 4:** Specification of the distances $l_{ij}$ in millimeter between the position of a marker and the adjacent rotational axes for the robot used. Note that only the translation is specified here, assuming a robot in rest position (all joint angles 0°, i.e. orientation is vertical upwards).

${\varvec{i}}$	${\varvec{j}} = 1$	${\varvec{j}} = 2$
$0$	$\left( {71.6, 0, - 24.6} \right)^{T}$	$\left( {0 , 0 , - 24.6} \right)^{T}$
$1$	$\left( { - 65.6 , - 5.5 , 0} \right)^{T}$	$\left( {58.7 , - 5.5 , 0} \right)^{T}$
$2$	$\left( { - 65.8 , - 5.5 , 0} \right)^{T}$	$\left( {58.8 , - 5.5 , 0} \right)^{T}$
$3$	$\left( { - 237.9 , - 1.8 , - 33.6} \right)^{T}$	$\left( {42.8 , - 5.5 , 0} \right)^{T}$

## Appendix: Figures

See Figs. 8, 9 and 10.

## Declarations

### Animal or human studies

This work does not involve animal or human studies.
