# Correction: Easy scalable, low-cost open-source magnetic field detection system for evaluating low-field MRI magnets using a motion-tracked robot

**DOI:** 10.1007/s10334-025-01258-y

**Published:** 2025-05-27

**Authors:** Pavel Povolni, Robin Bendfeld, Sergej Maltsev, Judith Samlow, Felix Glang, Praveen Iyyappan Valsala, Dominique Goerner, Dario Bosch, Sebastian Mueller, Florian Birk, Kai Buckenmaier, Klaus Scheffler

**Affiliations:** 1 High‑Field Magnetic Resonance Center, Max Planck Institute for Biological Cybernetics, 72076 Tübingen, Germany; 2 Institute for Nonlinear Mechanics, Department of Mechanical Engineering, University of Stuttgart, 70569 Stuttgart, Germany; 3 Department for Biomedical Magnetic Resonance, University of Tübingen, 72076 Tübingen, Germany

**Correction: Magnetic Resonance Materials in Physics, Biology and Medicine** **https://doi.org/10.1007/s10334-025-01239-1**

In the original version of this article, Tables 1 and 2 were missing and Tables 1 and 2 should have appeared as shown below.

**Table 1:** Overview of the different variants of shimming and the results achieved

	Motion Tracked Robot with self-developed Hall sensor			Cubic Robot with commercial Hall sensor	
	All 8 sensors	Interpolated 8 sensors	Just sensor H7	Measured data	Offset cor. data
Before shimming					
$B_{0}$ (mT)	45.373	45.377	45.456	42.947	43.068
$\Delta B_{0}$ (ppm)	12,784	8351	5870	9314	4642
After shimming					
$B_{0}$ (mT)	45.700	45.899	46.444	43.612	43.829
$\Delta B_{0}$ (ppm)	10,984	5828	2202	8372	2935
Reduction homogeneity after shimming (1 run) (%)	14.1	30.2	62.5	10.1	36.8
Reduction standard deviation after shimming (%)	25.9	8.9	65.9	14.3	37.9
Amount shimming magnets used	761	727	778	763	784

**Table 2:** List of the measured gradient values determined along the $x$-axis from the 4 measurements described above

	Mean value in mT/m/A (deviation from Simulation)	Variance $\sigma$ In mT/m/A	FOV size in mm (measured points)
Motion Tracked Robot with self-developed Hall sensor	1.902 (− 9.6%)	0.440 (0.284 if interpolated)	Cubic, 100 × 30 × 40 (288 points)
Cubic Robot with commercial Hall sensor	1.899 (− 9.8%)	0.245	Spherical, $\varnothing$ 60 (190 points)
MRI scan (3T)	2.271 (+ 8.2%)	0.051	Cubic, 20 × 20 × 20 (1000 points)
Simulation	2.085	0.008	Spherical, $\varnothing$ 40 (3023 points)

The FOVs differ in the measurements in order to use as many available points as possible for the individual gradient determination

The original article has been corrected.

