# Supplementary material for: Easy scalable, low-cost open-source magnetic field detection system for evaluating low-field MRI magnets using a motion-tracked robot
Source: MAGMA. 2025 Apr 5;38(4):695–714. doi: 10.1007/s10334-025-01239-1 (PMC12443900; doi:10.1007/s10334-025-01239-1)
Supplement: Supplementary file 1 — Supplementary file1 (DOCX 4732 KB) [file 10334_2025_1239_MOESM1_ESM.docx]

**Supplementary Information: For inclusion in the Special Issue on Open Science**

**Easy Scalable, Low-Cost Open Source Magnetic Field Detection System for Evaluating Low-Field MRI Magnets using a Motion Tracked Robot**

Pavel Povolni^1^*, Robin Bendfeld^2^, Sergej Maltsev^1^, Judith Samlow^1^, Felix Glang^1^, Praveen Iyyappan Valsala^1,3^, Dominique Goerner^1^, Dario Bosch^1,3^, Sebastian Mueller^1,3^, Florian Birk^1,3^, Kai Buckenmaier^1^, Klaus Scheffler^1,3^

^1^ High‑Field Magnetic Resonance Center, Max Planck Institute for Biological Cybernetics, 72076 Tübingen, Germany

^2^ Institute for Nonlinear Mechanics, Department of Mechanical Engineering, University of Stuttgart, 70569 Stuttgart, Germany

^3^ Department for Biomedical Magnetic Resonance, University of Tübingen, 72076 Tübingen, Germany

* Corresponding author. E-mail: pavel.povolni@tuebingen.mpg.de

# Preamble

The following section provides a comprehensive description of technical aspects of the Hall sensors and the built system, including the calibration process. It should be noted that the **Supplementary** **Information** employs a coordinate system integrated within the sensor, which differs from the coordinate system utilized in the main part of the paper. The most notable discrepancy is the different definition of the $y$- and $z$-axes. The coordinate systems are illustrated in Figure S1.


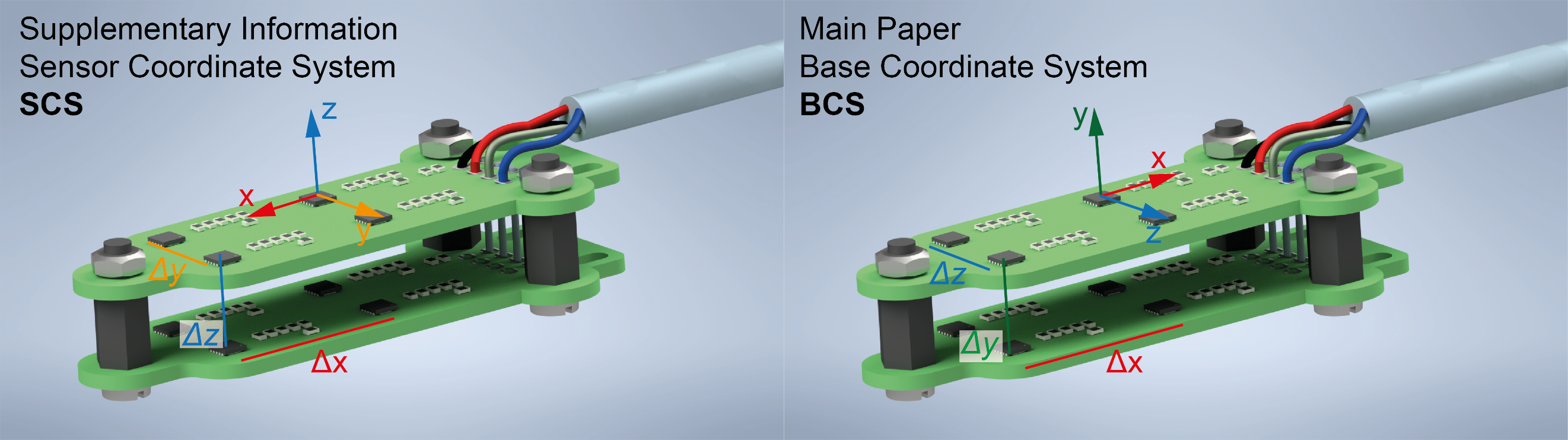


**Figure S1:** Schematic representation of the two coordinate systems used for the sensor (different use in the Supplementary Information and in the paper).

# Description of mapped Devices

## Halbach Low Field Magnet

The mapped Halbach magnet was build out of 536 magnets with a volume of $12x12x12mm^{3}$ and a magnetic field strength of $1.395T\pm25mT$ each (NdFeB, N48, Supermagnete, Gottmadingen, Germany). The magnets are distributed across 13 slices, whereby each slice consisted of two Halbach rings, with the outer ring containing four more magnets than the inner ring and positioned at a distance of $20mm$ from the inner ring. The distance between two slices is $22mm$. A genetic optimization algorithm according to [5] is implemented to determine the number of magnets per ring and the radius of the inner Halbach ring for each slice, with the objective of maximizing homogeneity within a spherical FOV with a diameter of $50mm$ in the center. The magnets are pressed into a plastic slice ($12mm$ thick), which is composed to two thinner adjacent sub-slices made out of $6mm$thick PMMA, that have been cut out with a laser cutter ($35W$-CO_2_ laser Ultra R9000, Universal Laser Systems, Scottsdale, Arizona, US). The double number of sub-slices increases the manufacturing effort, but the smaller distance between the laser head and the material during the cut means that the laser itself remains more focused and the cutting edge is right angled. Production-related tolerances of the two sub-slices create a tight fit for the magnet. This is particularly advantageous as the magnets themselves have a large tolerance of the side length ($\pm0.1mm$). PMMA does not tolerate mechanical stresses well and tears quickly. However, it was not observed that both plates tear at the same position, ensuring that the mechanical attachment of the magnets is always guaranteed. The slices are held together by 22 M8 threaded rods made out of brass. The entire Halbach array has a length of $300mm$, a diameter (including shimming trays) of $362mm$, and a bore size of $160mm$. For the purpose of shimming, additional magnets with a size of $5x5x5mm$ and $1.43T$ each (NdFeB, N50, Supermagnete, Gottmadingen, Germany) can be positioned on the exterior of the Halbach magnet on 14 rings in Halbach configuration ($k=2$) with a radius of $166mm$ at a distance of $12mm$ (divided into 6 shim trays). To compute the additional shimming field, a genetic algorithm according to [5, 7] was used. In total 840 potential positions for the additional shim magnets could be occupied, for which the orientation of the magnet could be varied among the following orientations $0^{\circ}$, $90^{\circ}$, $180^{\circ}$ or $270^{\circ}$ if it was assigned to a certain position. In the following, only the $B_{z}$ direction along the $B_{0}$ main direction is considered for shimming[7].

## X-Gradient

A three-axis gradient system based on the software package of Amrein et al. was designed for a spherical FOV of $40mm ADDIN ZOTERO\_ITEM CSL\_CITATION \{"citationID":"rXyvRUfB","properties":\{"formattedCitation":"[18]","plainCitation":"[18]","noteIndex":0\},"citationItems":[\{"id":310,"uris":["http://zotero.org/users/10289748/items/3Z66PGTL"],"uri":["http://zotero.org/users/10289748/items/3Z66PGTL"],"itemData":\{"id":310,"type":"article-journal","abstract":"Purpose An automated algorithm for generating realizable MR gradient and shim coil layouts based on the boundary element method is presented here. The overall goal is to reduce postprocessing effort and thus enable for rapid prototyping of new coil designs. For a given surface mesh and target field, the algorithm generates a connected, non-overlapping wire path. Methods The proposed algorithm consists of several steps: Stream function optimization, two-dimensional surface projection, potential discretization, topological contour sorting, opening and interconnecting contours, and finally adding non-overlapping return paths. Several technical parameters such as current strength, inductance and field accuracy are assessed for quality control. Results The proposed method is successfully demonstrated in four different examples. All exemplary results demonstrate high accuracy with regard to reaching the respective target field. The optimal discretization for a given stream function is found by generating multiple layouts while varying the input parameter values. Conclusion The presented algorithm allows for a rapid generation of interconnected coil layouts with high flexibility and low discretization error. This enables to reduce the overall post-processing effort. The source code of this work is publicly available ( https://github.com/Philipp-MR/CoilGen).","container-title":"Magnetic Resonance in Medicine","DOI":"10.1002/mrm.29294","ISSN":"0740-3194","issue":"3","journalAbbreviation":"Magnetic Resonance in Medicine","note":"publisher: John Wiley \& Sons, Ltd","page":"1465-1479","title":"CoilGen: Open-source MR coil layout generator","URL":"https://doi.org/10.1002/mrm.29294","volume":"88","author":[\{"family":"Amrein","given":"Philipp"\},\{"family":"Jia","given":"Feng"\},\{"family":"Zaitsev","given":"Maxim"\},\{"family":"Littin","given":"Sebastian"\}],"accessed":\{"date-parts":[["2024",5,26]]\},"issued":\{"date-parts":[["2022",9,1]]\}\}\}],"schema":"https://github.com/citation-style-language/schema/raw/master/csl-citation.json"\}$ [18], whereby the $x$-gradient, which changes $B_{0}$ along the cylindrical axis of the magnet, was mapped. The gradients were designed according to the target-field approach using streamline function with a winding pattern of 26 levels per quadrant. The coil was wound with $0.8mm$ thick insulated copper wire in a 3D-printed negative mold made of PETG. The $x$-gradient is $306mm$ long and has an inner diameter of $100mm$. The inductance and resistance were measured to $78.5\mu H$ and $0.68\Omega$, respectively. The gradient system is passively cooled. The gradient tube was rotated, such that its field is oriented in the $y$-direction of the BCS.

# Further information to the Hall Sensor

## Transmission speed of the used I²C-Prototcol

The maximum possible oversampling in a given measurement period is constrained by the update rate of the sensor (at maximum resolution approx. $2kHz$), the speed of the microcontroller (clock up to $160MHz$), and the speed of the used I²C-Bus. The latter is the limiting factor due to the parasitic capacitances on the bus lines. The data to be transmitted for a readout cycle of one Hall sensor in the I^2^C protocol consists of $102bits$ per sensor, resp. $816bits$ for one whole measurement because the eight sensors are read out sequentially. The speed of the I^2^C bus is largely determined by the parasitic capacitance on the bus lines and the power of the bus driver on both sides. All eight hall sensors, as well as the central microcontroller, are connected to the I^2^C data lines simultaneously, resulting in a total capacity of approximately $900pF$ to be reloaded[1]. The limiting factor for the I²C bus is the maximum power of the hall sensor bus driver (which is less than that of the microcontroller), which needs to recharge the bus line. This rise time thus determines the speed of the I^2^C bus. A bus speed of $100kHz$ (equivalent to a baud rate of $100kBd$ for I^2^C) can be achieved by selecting appropriate push-up resistor for the I^2^C bus.

## Calculating Hall Sensor Resolution

The core of every analog sensor with a digital output is an ADC. For an ideal ADC with $n$ bits resolution, the theoretical signal-to-noise ratio (SNR) limit due to quantization noise calculates to[2]:

|  | ${SNR}_{dB}=6.02n+1.76dB$ | (2.1) |
| --- | --- | --- |

A real ADC has additional noise sources that are summarized in the parameter referred as ENOB, which is smaller than $n$. Arithmetic averaging generally increases the SNR. However, the SNR increase necessitates a measurement with uniformly distributed noise (e.g., white noise) and free of systematic errors. An arithmetic averaging of 1000 sampled measuring points is implemented in the Hall sensor. This results in an increase in the $ENOB$ of:

|  | $\Delta ENOB= \frac{20\cdot\log\sqrt{1000}dB}{6.02dB}=5 bit$ | (2.2) |
| --- | --- | --- |

The ADC integrated in the Hall sensor has an $ENOB$ of $10bits$, thereby enabling the total resolution of $ENOB = 15bits$ to be achieved through the averaging, resulting in a theoretical resolution limit of $3\mu T$ within the measurement range of $\pm50mT$. This corresponds to a relative limit of $67ppm$ in our $45mT$ Low Field Magnet. The commercial Hall sensor Series 9900 with a 3-axis Hall probe ZOA99-3208 (F.W. BELL/MEGGIT, Christchurch, UK) has a relative limit of $350ppm$ in the same field. The Hall sensor developed is therefore very comparable with the commercial system

## More detailed look on Oversampling

For the most time-efficient data transmission, the sensors are read out sequentially. This involves setting the Hall sensor only once during the startup. During the readout, the measured values are retrieved as quickly as possible and temporarily stored in the RAM of the CPU. Once the data transfer is complete, the measured values are averaged. This mode enables the reading of eight Hall sensors, each with 1,000 measured values, in 6.5 seconds. The processed data are then sent to the PC via USB. Oversampling is beneficial for the mitigation of stochastically distributed noise, such as white noise or quantization noise. However, it does not provide a solution for the removal of constant error, including constant offsets. This implies that the following temperature-dependent relationship applies to the measured magnetic field $B_{\mathrm{meas}}(T)$ of a real Hall sensor in relation to the real magnetic field $B_{real}$, with a temperature-dependent sensitivity $a_{\mathrm{real}}(T)$ and offset $B_{OS}\left( T \right)$:

|  | $B_{\mathrm{meas}}\left( T \right)= a_{\mathrm{real}}\left( T \right)\cdot B_{\mathrm{real}}+B_{OS}(T)$ | (2.3) |
| --- | --- | --- |

The Hall sensor utilized is equipped with an integrated signal processing and temperature compensation, for which a simplification of the sensitivity with a 2^nd^ order Taylor expansion of the second order is sufficient . This approach was described in an Application Information by the same manufacturer for a similar Hall sensor[3]. For the offset, a Taylor expansion of the first order is used, as the Halbach magnet is mapped in a temperature stable environment. This leads to:

|  | $a_{\mathrm{real}}\left( T \right)=a_{\mathrm{real}}+a_{TC1}\cdot\left( T-T_{\mathrm{ref}} \right)+a_{TC2}\cdot\left( T-T_{\mathrm{ref}} \right)^{2}$ | (2.4) |
| --- | --- | --- |
|  | $B_{OS}\left( T \right)=a_{TC3}\cdot\left( T-T_{\mathrm{ref}} \right)$ | (2.5) |

with the sensitivity $a_{\mathrm{real}}$ and offset $B_{OS}$ of the sensor, and the temperature-dependent sensitivity coefficient $a_{TC1}$ and $a_{TC2}$ and offset coefficient $a_{TC3}$. The temperature dependence is related to a reference temperature $T_{\mathrm{ref}}=25^{\circ}C$, at which the Hall sensor is considered to be absolutely calibrated. For each axis per sensor, the real sensitivity and the temperature constants differ, as these originate from the actual measuring principle itself (imperfection of the semiconductor crystal, mechanical stress, piezoresistive effects etc.), even if they are partially compensated by the integrated signal processing[4].

# Calibration of the Hall sensor using a Solenoid as Reference Field

## Design Solenoid as Reference Magnetic Field

To facilitate easy reproducibility, the design of the solenoid coil for the reference magnetic field focused on the simplest possible available components. The central supporting tube has a diameter of $160mm$ and the length of the solenoid is $452.4mm$ (standardized HT DN160mm pipe with a length of $500mm$). The coil is wound from enameled copper wire with a diameter of $1.5mm$. The coil is composed of two layers: 290 windings on the inner layer and 27 windings on both end pieces in the outer layer. The number of outer turns was optimized numerically to maximize homogeneity in a FOV with a diameter of $10mm$ within the center of the coil. Since the FOV is located at a considerable distance from the conductors themselves, an analytical calculation can be performed. For this purpose, the cylinder is approximated as a superposition of adjacent conductor loops, using the analytical formula proposed by Simpson et al.[5]. For the purpose of simplicity, the solenoid was approximated as a parallel positioning of conductor loops. Consequently, the system is rotationally symmetrical. In cylindrical coordinates (with the radial distance from the center $\rho$ and the distance along the central symmetry axis $z$), the analytical formula then applies at one point[5] $(\rho|z)$:

|  | $B_{\rho}= \frac{\mu_{0}Iz}{2\gamma^{2}\beta\rho}\left[ \left( a^{2}+\rho^{2}+z^{2} \right)E\left( k^{2} \right)-\gamma^{2}K\left( k^{2} \right) \right]$ | (3.1) |
| --- | --- | --- |
|  | $B_{z}=\frac{\mu_{0}Iz}{2\gamma^{2}\beta}\left[ \left( a^{2}-\rho^{2}-z^{2} \right)E\left( k^{2} \right)+\gamma^{2}K\left( k^{2} \right) \right]$ | (3.2) |

With the constants:

|  | $\gamma^{2}=a^{2}+\rho^{2}+z^{2}-2a\rho$ | (3.3) |
| --- | --- | --- |
|  | $\beta^{2}=a^{2}+\rho^{2}+z^{2}+2a\rho$ | (3.4) |
|  | $k^{2}=1-\left( \frac{\gamma}{\beta} \right)^{2}$ | (3.5) |

with the current $I$, radius of the conductor loop $a$ and the elliptical integrals $E(k)$ and $K(k).$ The resulting current-dependent magnetic field is $780.7\mu T/A$ with a homogeneity of $2.4ppm$ in the FOV. The simulation results are shown in Figure S2a. This result can be verified by using the following equation, with which the magnetic field on the center of a solenoid along the $z$-direction can be calculated[6]:

|  | $B_{z}\left( z \right)= \frac{\mu_{0}nI}{2}\left( \frac{\frac{l}{2}-z}{\sqrt{R^{2}\left( \frac{l}{2}-z \right)}^{2}}+\frac{\frac{l}{2}+z}{\sqrt{R^{2}\left( \frac{l}{2}+z \right)}^{2}} \right)$ | (3.6) |
| --- | --- | --- |

with the current $I$, the number of turns $n$, the length $l$, and the radius $R$ of the designed coil.

The home-built coil used in this work consists of three overlapping cylindrical coils (one in the middle and two each at the ends), which can be calculated by superposition of the calculated three individual fields using (3.6). At the isocenter of the coil equation (3.2) and (3.6) yield the same result. It should be noted that the simple equation (3.6) does not permit any assertion to be made regarding the homogeneity within the FOV. FEM simulations (rotationally symmetric 2D simulations) with the open-source tool FEMM[7] confirm this result in the isocenter (deviation $-0.8\%$ to the analytical solution), for which the calculated results are shown in Figure S2b.


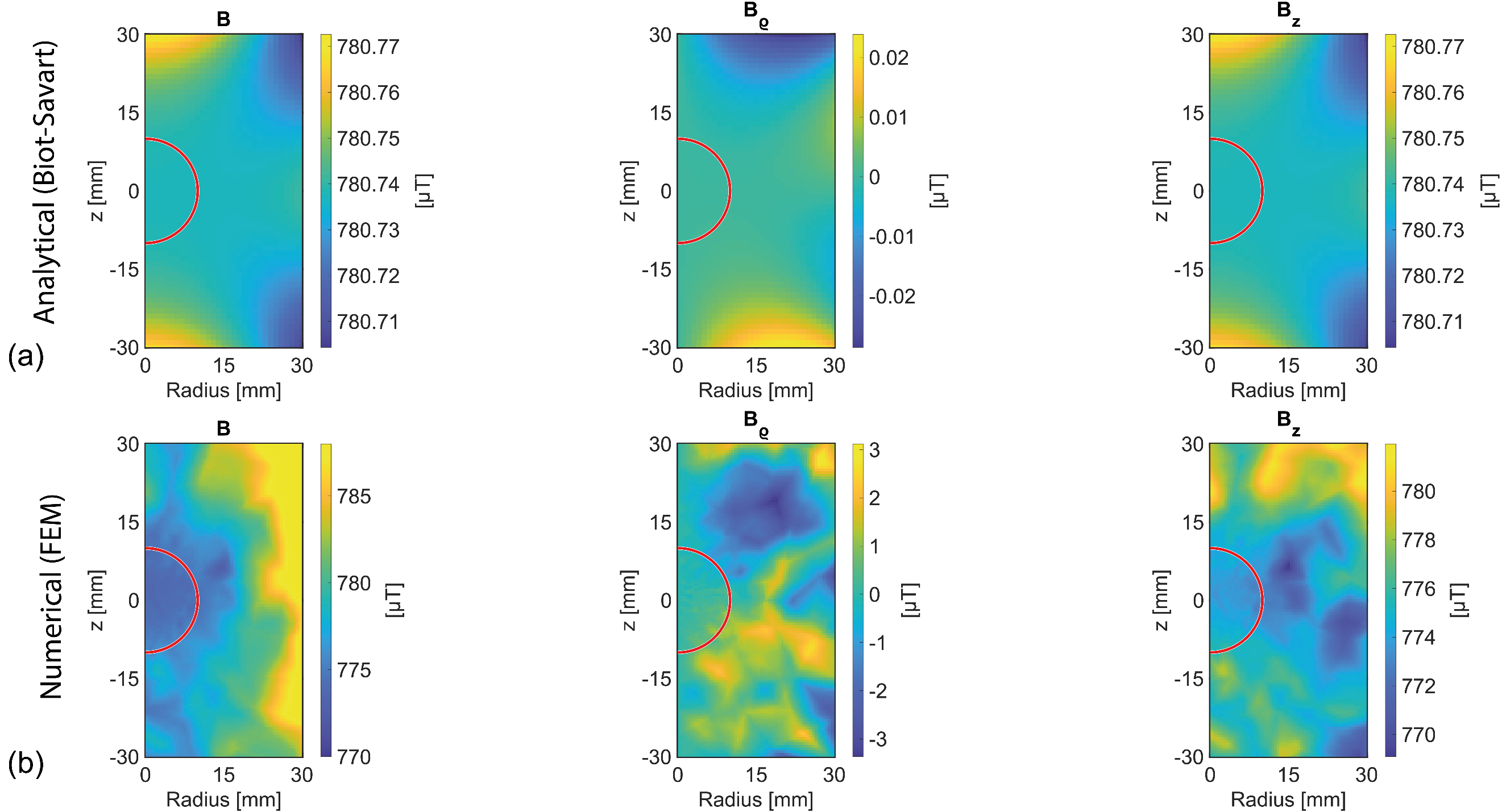


**Figure S2: a)** The analytically calculated rotationally symmetric magnetic field of the solenoid. On the left is the magnitude, in the center the component in the radial direction, and on the right the magnitude along the symmetry axis. The FOV in the center (red circle) has a radius of $10mm$. **b)** Numerically calculated magnetic field. On the left is the magnitude, in the center the radial component, on the right the component along the symmetry axis.

## Determination of the Error of the Solenoid used as a Reference Magnetic Field

In the analytical solution, the current flow is calculated for an ideal current path of perfectly parallel arranged conductor loops. In the FEM analysis, the spatial expansion of the copper conductors is considered, but the conductor loops are also perfectly parallel to each other due to the rotation symmetry. In the real cylinder, the conductor loops are slightly tilted relative to the axis of rotation, and the positioning of the conductor loops cannot be perfectly maintained, particularly in the second plane. This inevitably leads to a deviation in the real solenoid, which can only be determined using precise sensors, like NMR-based sensors.

In our case we analyzed the build solenoid in a commercial 3T MRI scanner (Prisma, Siemens Healthineers, Erlangen, Germany) using a 1-liter water bottle as a phantom. A current of $260mA$ was applied to the solenoid. During the measurement the body coil was used for transmit, and a 1-channel Flexcoil inside the solenoid was used for receive. A double spin-echo sequence with $\Delta T_{E}=3.8ms$ was used to measure the generated $B_{0}$ inhomogeneity. A voxel-size of $1.72x1.72x2.0mm (XYZ)$and a readout bandwidth $450 Hz/px$ was set. The magnetic field of the solenoid was oriented parallel to $B_{0}$, such that the spatially varying magnetic field of the solenoid overlapped with the read gradient (along the $z$-axis of the magnet). Since this additional magnetic field was not considered during encoding or reconstruction of the MRI scan, the bottle appeared shifted along the $z$-axis in the acquired image. By measuring this offset the magnetic field of the coil can be calculated. It should be noted that a new frequency adjustment of the MR scanner is tricky , as it does not only affect the small homogenous center of the solenoid. The determined $B_{0}$-maps are shown in Figure S3 for the middle plane along the $z$-direction. A large, homogeneous area in the center of the coil is clearly visible, indicating that the positioning of the Hall sensors does not have to be particularly precise during calibration procedure. The shift along the $y$-direction of the plot due to the magnetic field of the solenoid was analyzed using the $B_{0}$-maps in Figure S4, which are slightly off-center. It should be noted that this analysis is only approximate, as no fixed geometric structures within the phantom are available for analysis. Accordingly, inhomogeneities resulting from the bottleneck were selected for analysis. Two exemplary slices in the $xy$-plane demonstrated a shift of $\Delta y=18\ldots19px$. This corresponds to a frequency shift of $\Delta f=8100\ldots8550Hz$, resp. to a magnetic field of $190.2\ldots200.8\mu T$ in the isocenter. Consequently, a current-dependent magnetic field of $731.5\ldots772.3\mu T/A$ is produced, or a deviation of $-6.7\ldots-1.1\%$ compared to the simulation. It should be noted that the calculated value is also subject to a potentially unknown measurement error. This might for example be due to the influence of the read gradient, which can couple inductively to the solenoid, and the distortion of the MRI image due to the metals around the phantom. It is therefore not feasible to determine the absolute homogeneity within the FOV from the acquired $B_{0}$ maps. However, as can be seen in Figure S3, the magnetic field is largely homogeneous within the FOV. Therefore, the exact positioning of the Hall probe in the isocenter during calibration is not a significant factor.





**Figure S3:** The measured $B_{0}$-map in a 3T-MRI-scanner, with the solenoid in a state with no current (left) and with a current of $260mA$ switched on (right), reveals a shift of the phantom in the negative y-direction due to the switched-on current.


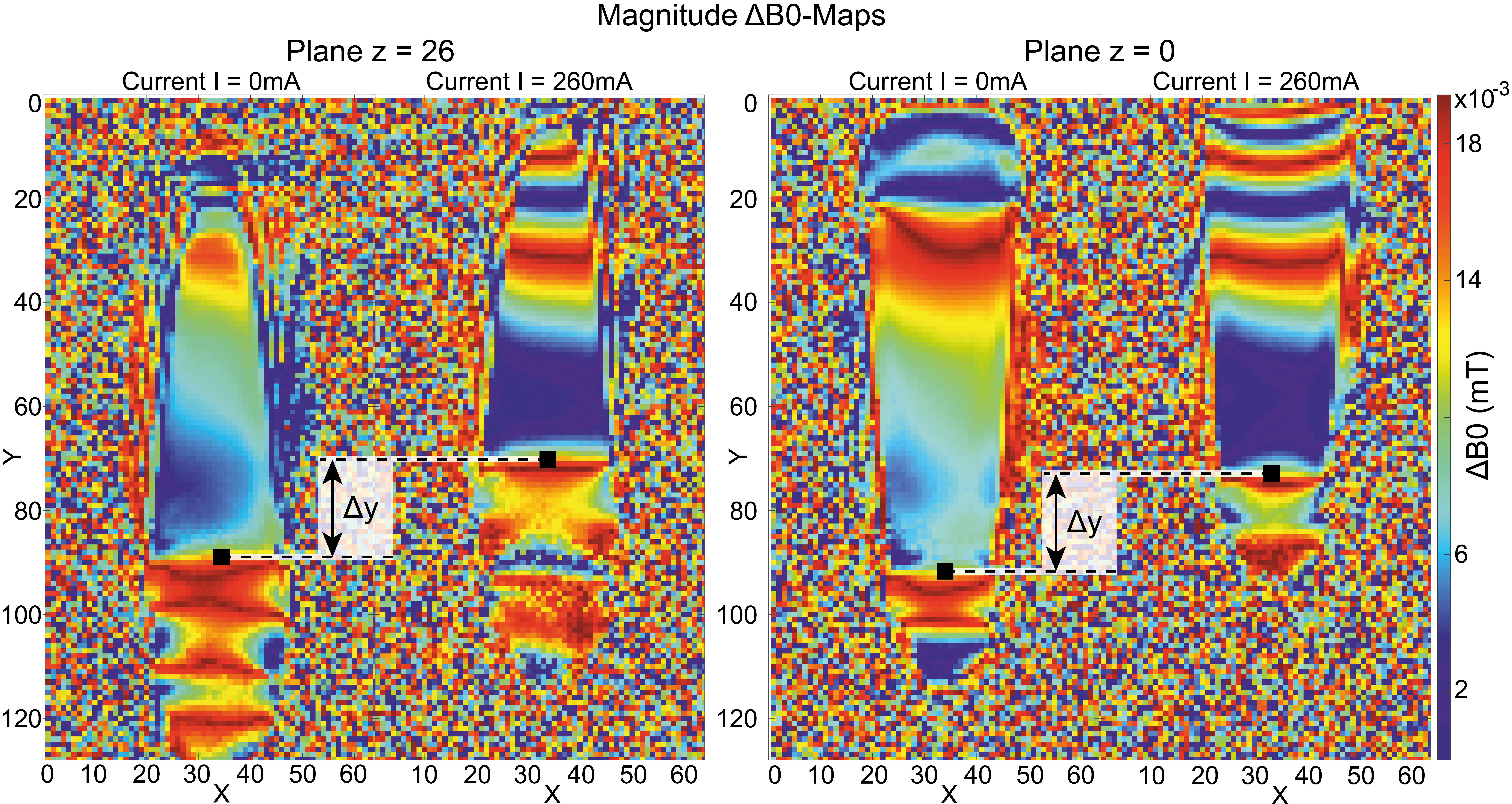


**Figure S4:** The measured B0 map used to determine the magnetic field strength of the solenoid

In the end, a minor optimization was implemented as a solution to determine the deviation of the solenoid in post-processing. An additional error parameter was included in the calibration algorithm, which was used to determine the deviation of the magnet current factor. The calibration of the sensors was determined for various error parameters. Depending on the error parameter, this results in an individual calibration of all eight sensors, in which the calculated sensitivities for all three axes deviate from the data sheet value. The error parameter that results in the smallest cumulative deviation from the data sheet values across all eight sensors and three axes is determined and used in the following as the total error. This results in a deviation of the actual cylinder from the simulation of $-4.2\%,$ which is consistent with the MRI measurement.

The strategy used to determine the error of the solenoid without the use of expensive additional equipment is highly effective for the $x$- and $y$-axes of the hall sensor, which are the most precise in the sensor due to their planar design in the semiconductor. However, the $z$-axis present challenges, with some Hall sensors showing sensitivities that exceed the data sheet specifications. In principle, the $z$-axis is also the least sensitive, due to the fact that the Hall volume passes vertically through several silicon layers, resulting in geometric deviations. However, this only plays a minor role in the end, as it is demonstrated in the field mapping of the magnet itself.

## Cooling

In order to achieve an optimal calibration, it is necessary to cover as much as possible of the sensor range, which necessitates the use of high currents (lower number of windings) or high voltages (higher number of windings). Concurrently, a balance must be found for the number of windings to prevent the generation of excessively high voltages (possible danger to life) or high losses within the solenoid (possible danger to the system). Therefore, higher currents are preferred, which can be readily and safely generated using standard batteries commonly employed in model-making or as automobile parts. The built solenoid has a resistance of $1.6\Omega$, which results in losses of $1.6kW$ for a field strength of $25mT$ (resp. a current of ca. $32A$). The measurement of a single measuring point only takes a short time and therefore a small dissipated energy loss, thus allowing for the design of the calibration cylinder to be greatly simplified with a sufficiently large passive cooling system. In this configuration, a second tube with an outer diameter of $200mm$ (standard HT DN200 pipe with a length of $500mm$) is positioned over the cylinder coil, with the tubes being firmly sealed together. The resulting cavity of approx. $3.4l$ is filled with water. Under the assumption that the entirety of the power loss of the cylinder is transferred to the cooling water and not released into the environment (a worst-case scenario), the temperature increase can be calculated using the following equation:

|  | $\Delta T=\frac{P\cdot t}{m\cdot c}$ | (3.7) |
| --- | --- | --- |

with the ohmic power loss $P$, the duration $t$, the mass of the water $m=3.4kg$ and the heat capacity of water $c=4190Ws{kg}^{-1}K^{-1}$. Assuming that the entirety of the power loss of $1.6kW$ (at $25mT$) is stored in the water and no energy is radiated, the water will heat up by approximately $0.7K$ during a measurement of 6.5 seconds. The system is capable of being heated to a temperature of up to $80^{\circ}C$, which allows for a sufficient number of measurements to be conducted or before the water needs to be replaced.

## Setup of the calibration measurement/ Placing the sensor inside the solenoid

The Hall sensor can be positioned in the center of the cylinder using a fixture. Due to the rotational symmetry of the cylinder, parallelism is not a concern. The homogeneous calibration cylinder permits the simultaneous measurement of two neighboring Hall sensors. Given that the magnetic field of the cylinder is oriented along its symmetry axis, it is necessary to carry out the calibration separately for each axis. The current in the cylinder is measured via a shunt with $20m\Omega$ ($0.1\%$tolerance, $\pm1ppm/K$, RUG-Z-R020-0.1-TK1, Isabellenhütte Heusler, Dillenburg, Germany) using a Kelvin measurement and a multimeter (GDM-8255A GW-Instek, Taipeh City, Taiwan). By measuring the DC current the magnetic field in the center can be determined.

In order to record more measured values for a linear regression, the number of averages on the Hall sensor itself was reduced to 50. Three laboratory power supplies (EA-PS 3065-10B, Elektro-Automatik, Viersen, Germany) are operated in parallel as current sources, allowing to achieve currents up to $30A$. Higher currents were achieved with eight series-connected $12V$-lead-acid batteries (HRL1234W, CSB Energy Technology, Taipeh City, Taiwan; $R_{i} = 17mΩ$, discharge current $130A$ for $<5s$). A maximum current of up to $43A$ (equal to $32.8mT$) was achieved due to bad ohmic connection at the battery terminal. For each current set, 10 measured values were recorded, with the current increased in 13 steps up to a maximum field of $32mT$. The polarity was then reversed, and the measurement repeated. This yields 26 measured values for the linearity measurement over a range of $-32$ to $32mT$.

Due to ohmic losses, the coil heats up and thus heats the surrounding air and the sensor head itself. In order to achieve the temperature correction in the calculation of the linearization as effectively as possible (see equation (2.4) and (2.5)), the temperature of the sensor head is increased to a maximum of $40^{\circ}C$using a hot air gun after the linearization measurement at a constant field of approximately 2$0mT$. This temperature compensation is not necessary in the subsequent measurement in the Halbach magnet as long as the room temperature is kept constant (which is typically the case). In this temperature measurement, all eight sensors were measured simultaneously, resulting in an error due to the homogeneity of the coil. This error is considered by a weighting factor in the evaluation of the measurement results. It should be noted that these measurements are carried out in an unshielded laboratory. This implies that the sensors are exposed to the earth's magnetic field and potentially to the effects of stray fields from neighboring machines. In order to ensure consistency, the calibration coil was identically positioned and aligned for each measurement. Furthermore, the sensors were installed in the same direction along the magnetic field of the coil, ensuring a consistent approach to measuring across all three axes. Therefore, the constant offset error is consistent across all measurements. Furthermore, the absolute offset of the eight Hall sensors was quantified. Access to a magnetically shielded chamber was possible (VACOShield, Vacuumschmelze, Hanau, Germany) which ensures a residual field of $<10nT$ inside[8]. The sensor was positioned within the chamber and $1,000$ measurement points were recorded, which were used for this calibration. At the same time, the calibration software is designed in a way to determine this offset automatically.

## Measurement Error

Measurement errors in the calibration setup have a direct effect on the quality of the calibration and determine the maximum resolution. The span of the measurement error can be calculated using the following equation:

|  | $g_{\mathrm{Coil}}\cdot\frac{U_{\mathrm{Multi}}-\Delta_{\mathrm{Multi}}}{R_{\mathrm{Shunt}}+\Delta_{\mathrm{Shunt}}}\leq B_{\mathrm{Coil}}\leq g_{\mathrm{Coil}}\cdot\frac{U_{\mathrm{Multi}}+\Delta_{\mathrm{Multi}}}{R_{\mathrm{Shunt}}-\Delta_{\mathrm{Shunt}}}$ | (3.8) |
| --- | --- | --- |

with the conversion factor of the calibration coil $g_{\mathrm{Coil}}$, the measured voltage with the multimeter $U_{\mathrm{Multi}}$, the measurement error of the multimeter $\Delta_{\mathrm{Multi}}$, the shunt resistance $R_{\mathrm{Shunt}}$ and the error of the shunt $\Delta_{\mathrm{Shunt}}$. The measurement error of the multimeter $\Delta_{Multi}$ (systematic error) comprises an offset $\Delta_{M,OS}$, a sensitivity error $\Delta_{M,Sen}$ and a non-linearity error $\Delta_{M,Lin}$:

|  | $\Delta_{\mathrm{Multi}}=\vert\Delta_{M,OS}\vert+\vert\Delta_{M,Sen}\vert+{\vert\Delta}_{M,Lin}\vert$ | (3.9) |
| --- | --- | --- |

As an example, an error of $\Delta_{M,OS}=50\mu V$, $\Delta_{M,Sen}=65.5\mu V$, and $\Delta_{M,Lin}=786.2\mu V$ was observed with the multimeter utilized at a field of $25mT$ ($32.758A$, which corresponds to $655mV$ using the shunt). The shunt was mounted on a heat sink in the setup, for which a temperature rise could not be detected. Consequently, the error of the shunt is reduced to the manufacturing tolerance of $0.1\%.$ In total these errors result in an error margin of $118\mu T$ in the center for a magnetic field of $25mT$ ($0.5\%$). It should be noted that a comparatively precise shunt has been selected in this setup. Cheaper and less precise resistors can also be selected and the real resistance can be determined with an precise conventional resistance measurement before starting the measurement.

## Result of the Measurement

The recorded values are evaluated in MATLAB during post-processing. An optimization-based algorithm is used to determine the coefficients from equation (2.4) and (2.5) for each sensor and each axis. The objective of the optimizer is to fit the measured values as closely as possible to the target values, while keeping the sensitivity and temperature dependence inside the datasheet limits.

All of the measured data (obtained from the sensitivity and temperature dependence measurements) were utilized collectively for the fitting process. The offset determined inside the shielding chamber (974 measured points for all sensors) was averaged and incorporated into the measured values prior to optimization. The optimization procedure involves running through all eight sensors independently, with all three axes optimized simultaneously for each sensor.

In the cost function, the sum of the squared difference between the magnetic field of the calibration coil and the optimized measurement of the Hall sensors is calculated for each axis of the sensor. The three calculated sums are then weighted and added together (sensitivity measurement to temperature measurement in a ratio of 2:1). Prior to optimization, the data set is reviewed to identify and remove all measured magnetic field values at temperatures exceeding $40^{\circ}C$ (the approximate temperature dependence in equation (2.4) and (2.5) only applies in a limited temperature range).

Prior to optimization, the data set is divided into a training set and a test set in a ratio of 2:1. This split is performed by selecting every third element for the test set. Summing up all 8 Hall sensors results in a data set with the average mean of elements of 565.5 for the $x$-axis, 741 for the $y$-axis, and 666.5 for the $z$-axis.

An exemplary calibration is shown for the $y$-axis of Hall sensor H7 in Figure S5a. In these figures, the training data set (on which optimization was performed) and the test set are shown separately. It is evident that the error for this Hall sensor is smaller than the measurement error itself. It can therefore be assumed that the calibration was successful. Figure S5b shows the standard deviation of the difference between the reference field and the calibrated Hall sensor, which is calculated by:

|  | $STD= std\left( B_{\mathrm{Coil}}- B_{\mathrm{meas}}(T) \right)$ | (3.10) |
| --- | --- | --- |

with $B_{\mathrm{Coil}}$ as the known magnetic field of the calibration coil, and $B_{\mathrm{meas}}(T)$ from equation (2.3) with the determined parameters. $STD$ is calculated for all eight Hall sensors and separately for the three axes, and also separated into a training set and test set. For all sensor axes, the $STD$ decreases after calibration compared to the same data set without a calibration. In particular, the calculated $STD$ after calibration of the test set are smaller or at least similar to those of the training set used. This indicates a successful calibration, allowing the assumption that the parameters determined for the equation (2.3) are also sufficiently accurate for subsequent measurements. In some cases, the optimized results are not better than the raw result. This indicates that this specific hall sensors are functioning almost optimally due to the manufacturing process, or that the measurement inaccuracy is already significant during the measurement and no further improvement can be achieved.


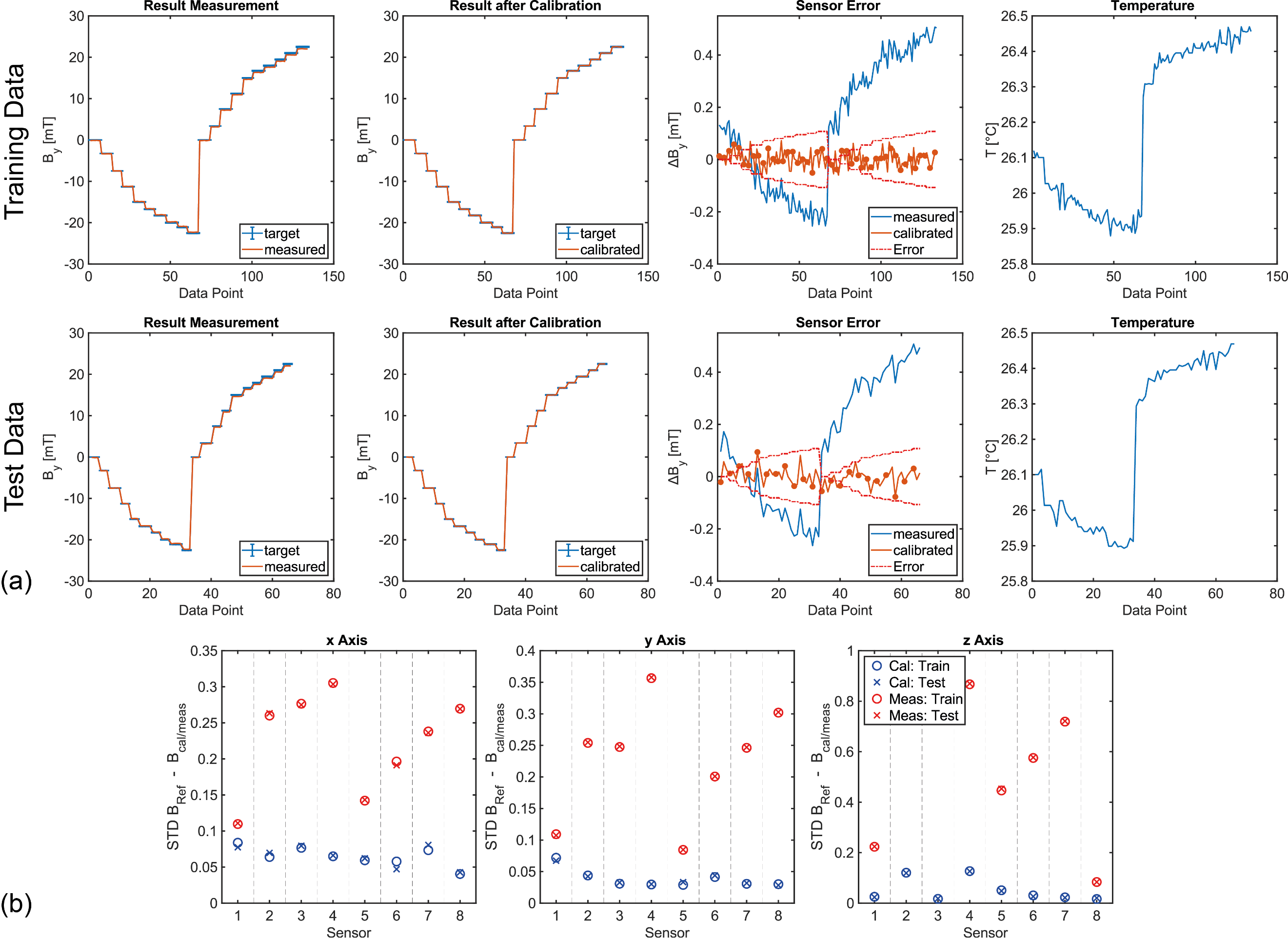


**Figure S5: a)** Shown is the data set for the y-axis of sensor H7, split in to a training and an test dataset. The original measurement is shown on the left, the successful calibration next to it. The sensor error prior to the calibration and after the applied calibration is shown in the third plot, while the measurement error is shown in dashed red. The error after calibration is consistently lower than the measurement error, indicating that the calibration was successful. The temperature curve of the measurement is shown on the right. **b)** The calculated standard deviation, as defined by equation (3.10), is plotted separately for each of the eight sensors and for each of the three spatial directions.

# References

1. Allegro Microsystems (2023) 3D Linear Hall-Effect Sensor with I2C Output and Advanced Low Power Management. Manchester, USA

2. Kester W, Bryant J (2004) Chapter 2: Fundamentals of Sampled Data System. The Data Conversion Handbook

3. Lapomardo J (2021) Calculating Temperature Compensation Using A1342 Linear Hall-Effect Sensor ICs. Allegro Microsystems, Manchester, USA

4. Popović RS (1989) Hall-effect devices. Sensors and Actuators 17:39–53.

5. Simpson J, Lane J, Immer C, Youngquist R (2001) Simple Analytic Expressions for the Magnetic Field of a Circular Current Loop.

6. Callaghan EE, Maslen SH (1960) The Magnetic Field of a Finite Solenoid. NASA Technical Reports NASA-TN-D-465 (E-900):

7. Meeker DC Finite Element Method Magnetics.

8. Buckenmaier K, Rudolph M, Fehling P, Steffen T, Back C, Bernard R, Pohmann R, Bernarding J, Kleiner R, Koelle D, Plaumann M, Scheffler K (2018) Mutual benefit achieved by combining ultralow-field magnetic resonance and hyperpolarizing techniques. Review of Scientific Instruments 89:125103.
