# Supplementary figures and images for: Easy scalable, low-cost open-source magnetic field detection system for evaluating low-field MRI magnets using a motion-tracked robot
Source: MAGMA. 2025 Apr 5;38(4):695–714. doi: 10.1007/s10334-025-01239-1 (PMC12443900; doi:10.1007/s10334-025-01239-1)

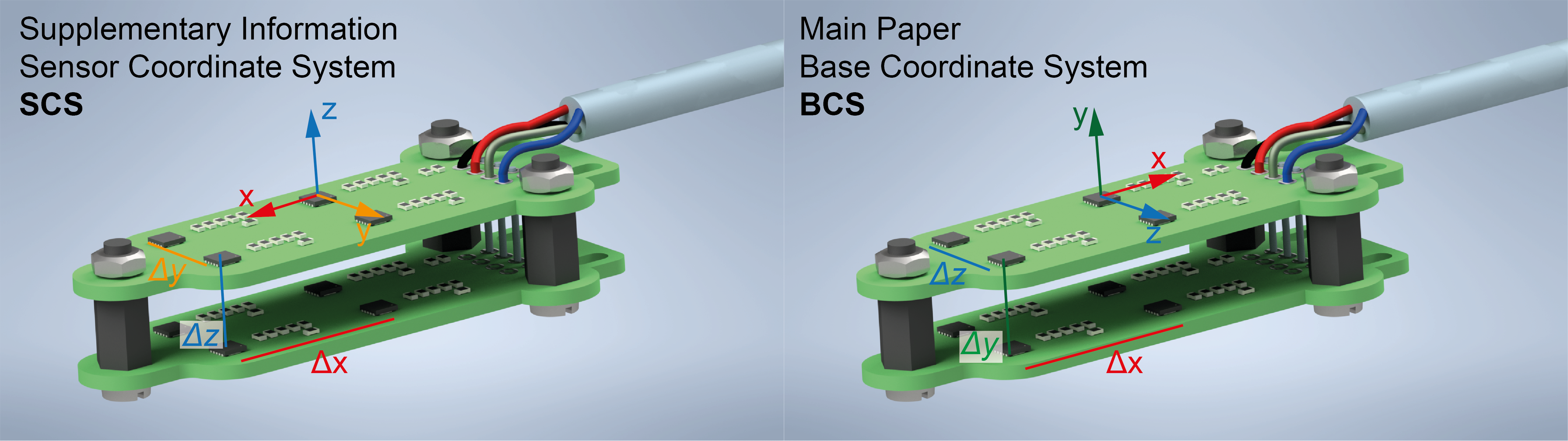

Supplement: Supplementary file 2 — Supplementary file2 (TIF 13608 KB) [file 10334_2025_1239_MOESM2_ESM.tif]

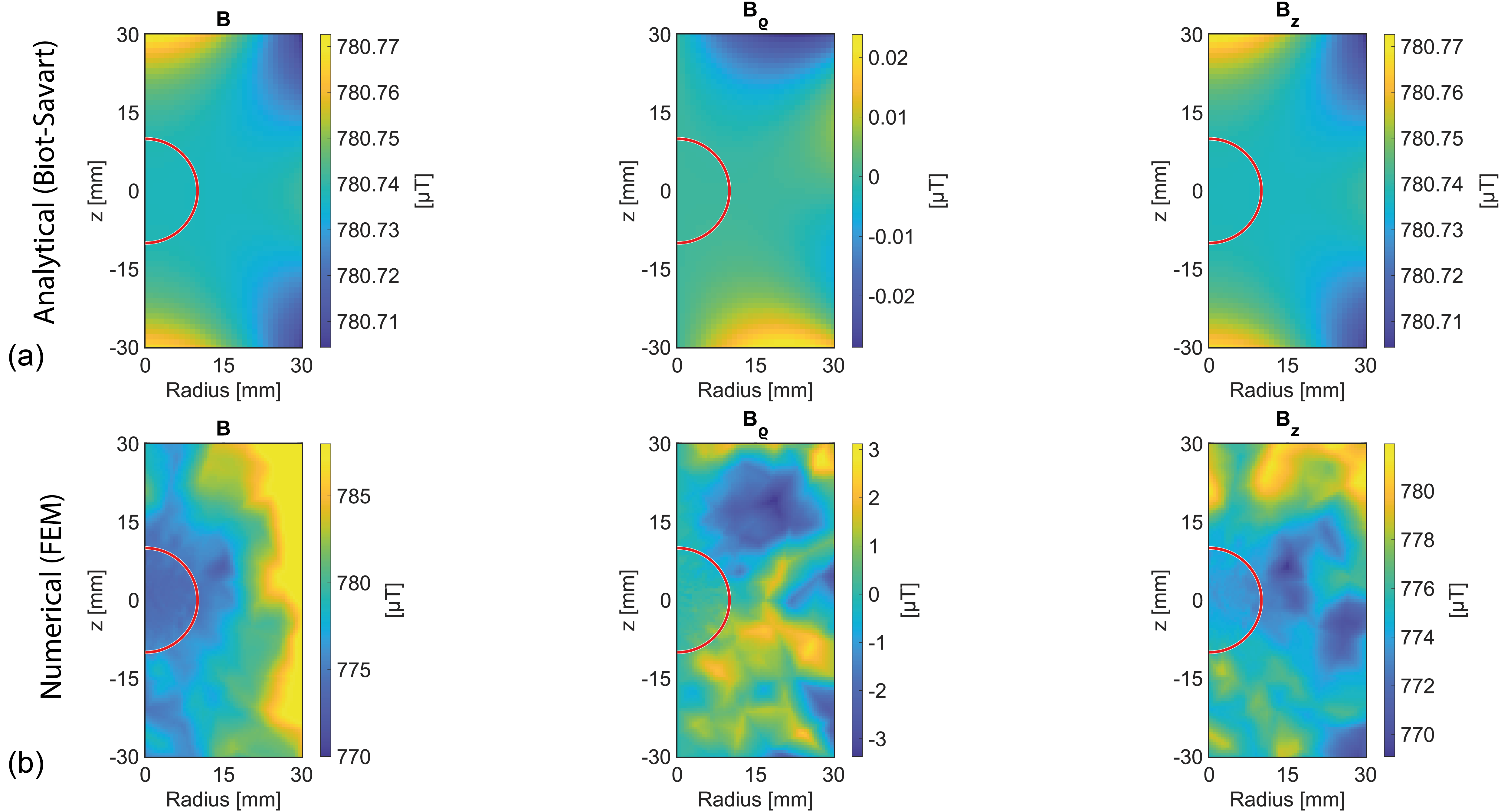

Supplement: Supplementary file 3 — Supplementary file3 (TIF 5149 KB) [file 10334_2025_1239_MOESM3_ESM.tif]

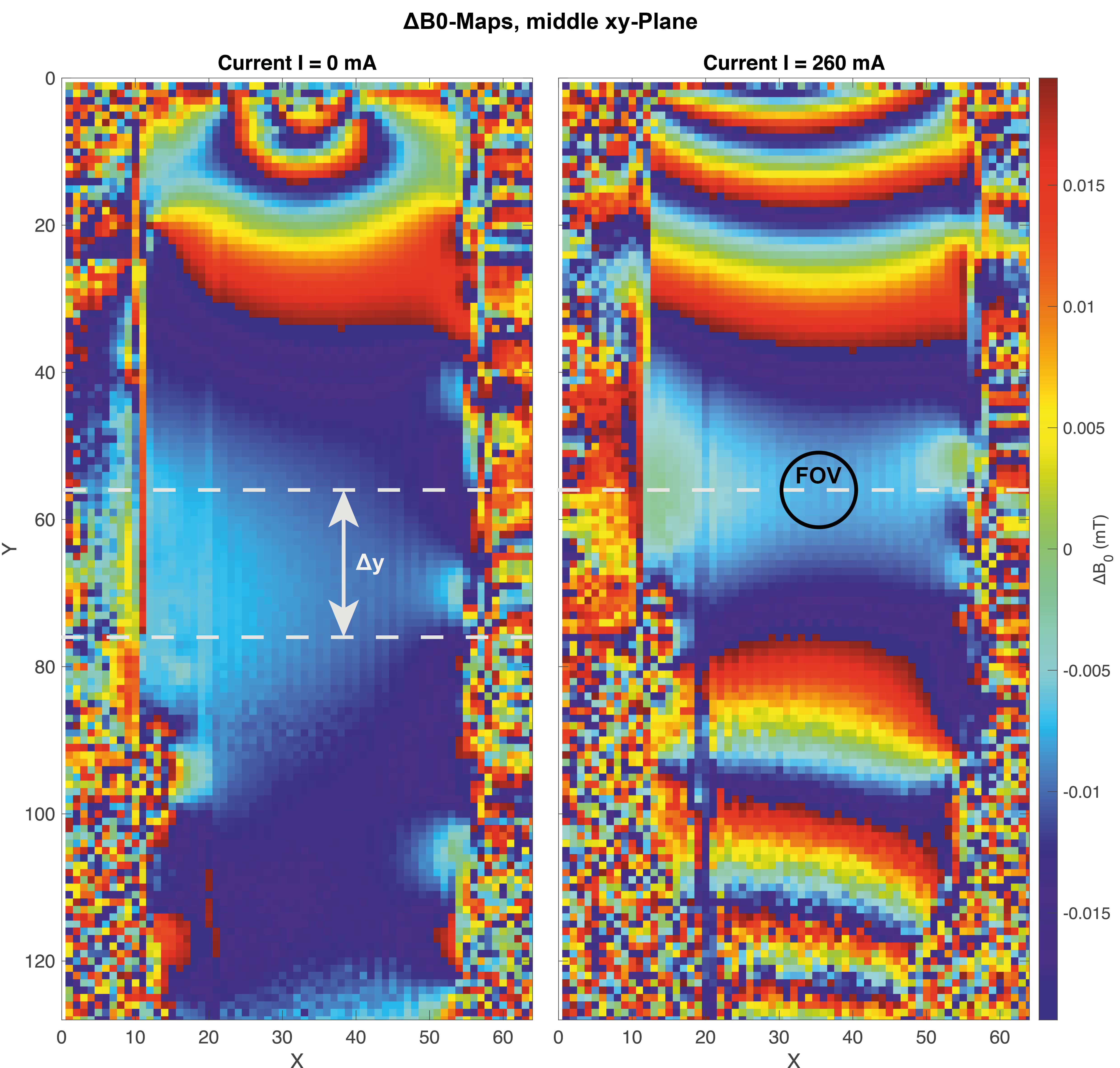

Supplement: Supplementary file 4 — Supplementary file4 (TIF 8060 KB) [file 10334_2025_1239_MOESM4_ESM.tif]

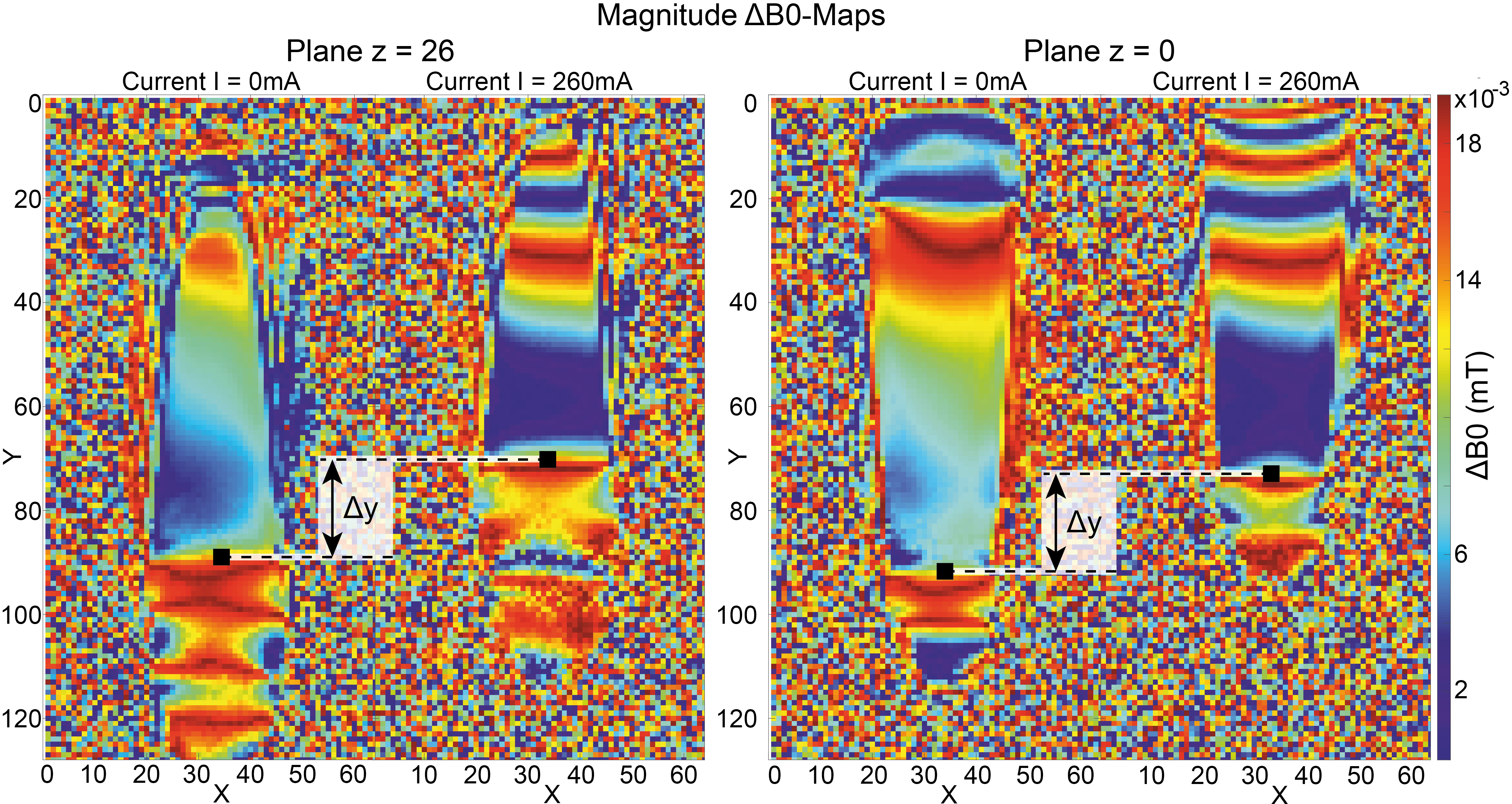

Supplement: Supplementary file 5 — Supplementary file5 (TIF 7693 KB) [file 10334_2025_1239_MOESM5_ESM.tif]

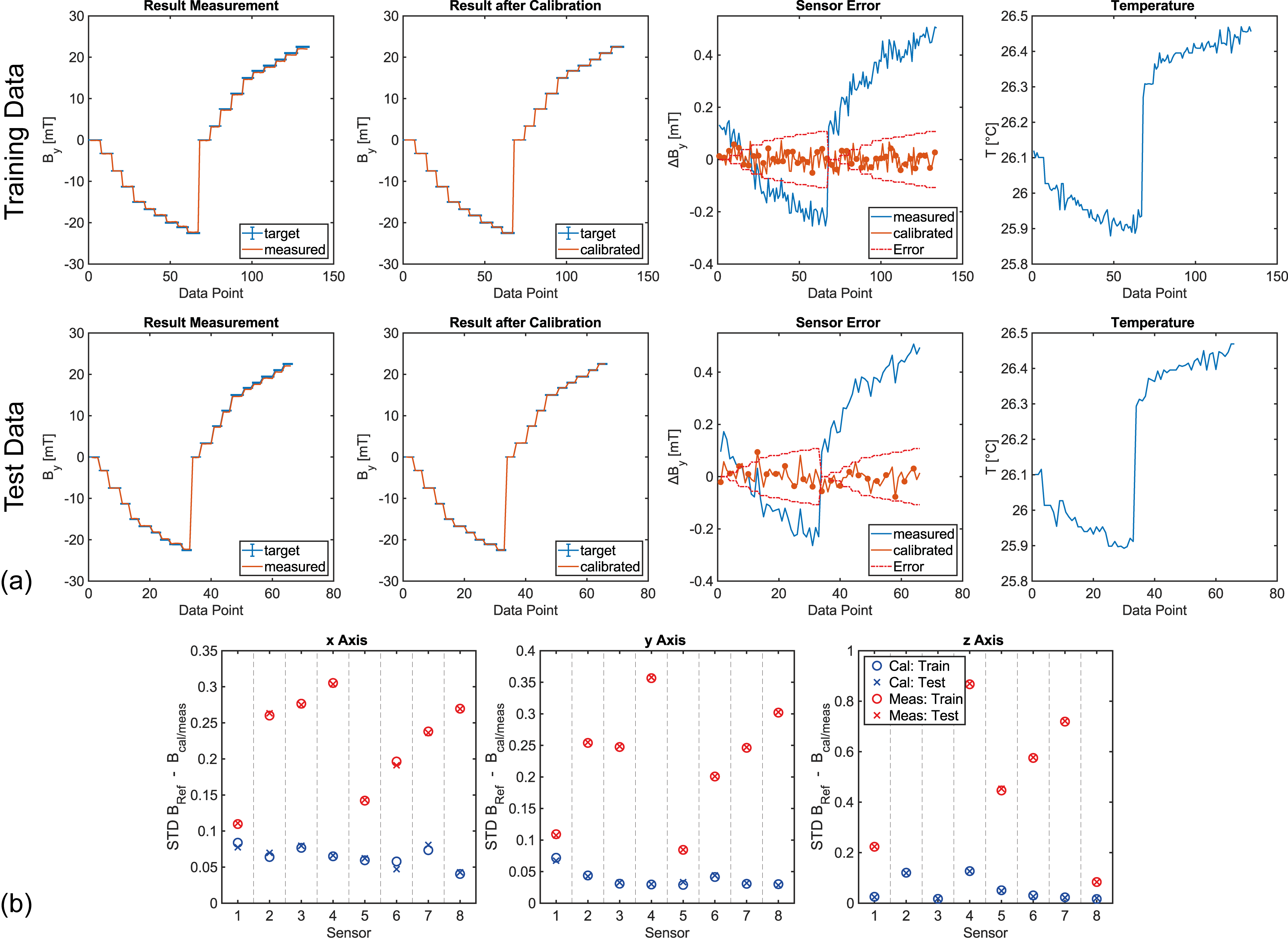

Supplement: Supplementary file 6 — Supplementary file6 (TIF 4539 KB) [file 10334_2025_1239_MOESM6_ESM.tif]
